# Effects of temperature fluctuations on quality deterioration and flavor evolution of pre-packaged tofu during cold-chain logistics

**DOI:** 10.3389/fnut.2026.1976213

**Published:** 2026-09-30

**Authors:** Dan Zhao, Jie Wu, Chengjun He, Yin Cao, Qiuli Zhong, Hao Chen, Zhanrui Huang, Le Xie, Liu Fan

**Affiliations:** 1 College of Economics and Management, Shaoyang University, Shaoyang, China; 2 Hunan Provincial Key Laboratory of Soybean Products Processing and Safety Control, Hunan Engineering Research Center of Green Processing and Equipment of Hunan-Style Food, Shaoyang, China; 3 College of Food Science and Chemical Engineering, Shaoyang University, Shaoyang, China; 4 Hunan Xianrun Biotechnology Co., Ltd., Shaoyang, China; 5 Yanjinpuzi Food Co., Ltd., Changsha, China

**Keywords:** cold chain logistics, flavor evolution, pre-packaged tofu, spoilage metabolism, temperature fluctuation

## Abstract

**Introduction:**

To investigate the effects of temperature fluctuation during cold chain logistics on quality deterioration and flavor evolution of pre-packaged tofu.

**Methods:**

This study simulated stable cold chain (CK) and two temperature fluctuation modes (T1 and T2), and systematically analyzed the microbial changes, spoilage metabolism, water status, texture properties, and volatile flavor composition of tofu under different storage conditions.

**Results:**

The results showed that temperature fluctuation significantly accelerated the quality deterioration of pre-packaged tofu, with T1 treatment exhibiting the most notable promoting effect. Compared with the CK group, temperature fluctuation promoted the increase of total viable count and psychrotrophic bacterial count, while also accelerating the accumulation of total volatile basic nitrogen (TVB-N), thiobarbituric acid-reactive substances (TBARS), and spoilage-related biogenic amines. After 35 days of storage, total bacterial count of the T1 group reached 6.52 lg CFU/g, and the contents of TVB-N and TBARS increased to 7.20 mg/100 g and 0.726 mg/100 g, respectively. Moreover, temperature fluctuations reduced the water-holding capacity of tofu, altered water distribution, and decreased hardness, springiness, chewiness, and resilience. Low-field nuclear magnetic resonance (LF-NMR) and magnetic resonance imaging (MRI) analyses further indicated that temperature fluctuations changed the internal water distribution of tofu. Electronic nose, gas chromatography-ion mobility spectrometry (GC-IMS) and gas chromatography-mass spectrometry (GC-MS) analyses showed that temperature fluctuations reshaped the volatile flavor profile of tofu and promoted the formation of aldehydes, acids, and nitrogen-containing volatile compounds.

**Conclusion:**

In summary, temperature fluctuations during cold-chain storage were associated with greater microbial proliferation, lipid oxidation-related changes, altered water distribution, texture deterioration, and pronounced changes in the volatile flavor profile of pre-packaged tofu. This study provides the theoretical basis to optimize cold chain transport conditions and the quality control of tofu.

## 1 Introduction

Tofu is a traditional plant-based protein food made from soybean protein gel. Due to its richness in high-quality protein, unsaturated fatty acids, minerals, and multiple functional components (1), it has a broad consumption base and significant industrial value in Asia, especially in China. In recent years, with the increase of consumer health awareness and the growing demand for plant-based foods, the scale of tofu and its related soy product industry has expanded, and the scope of product circulation has gradually increased (2). However, tofu has high moisture content and rich nutritional composition, with its internal three-dimensional gel network structure formed by soybean proteins providing a suitable environment for microbial growth and biochemical reactions. This makes it susceptible to microbial proliferation, protein degradation, lipid oxidation, and water migration during storage and circulation, which further lead to quality problems such as decreased water-holding capacity, texture deterioration, and flavor abnormality (3). Therefore, the effective quality deterioration control of tofu during circulation and its extended commercial shelf life has become an important issue that urgently needs to be addressed in soy product industries.

Currently, low-temperature storage, packaging technologies, and natural preservatives have been widely used to delay the deterioration of tofu and related soybean products (2, 4, 5). However, foods generally undergo multiple stages between production and consumption, including storage, transportation, transfer, and retail. Owing to changes in ambient temperature, refrigeration equipment operation, and loading and unloading practices, maintaining a constant temperature throughout the cold chain storage is difficult, and temperature fluctuations of varying degrees are therefore inevitable (6). In recent years, the impact of temperature fluctuations on the quality of foods stored at low temperatures has gradually attracted attention. Liu et al. (7) found that temperature fluctuations during cold chain storage induced water migration and tissue structure damage in *Litopenaeus vannamei*, leading to decreased water-holding capacity; Chen et al. (8) further pointed out that temperature fluctuations could alter the microbial community composition of large yellow croaker (*Pseudosciaena crocea*) filets, accompanied by changes in amino acid metabolism and enhanced protein degradation; Yin et al. (9) also demonstrated that temperature fluctuations during frozen storage could change the taste-active compound composition of tilapia filets, affecting their flavor quality. These studies indicate that, although foods are generally maintained at low temperatures, temperature fluctuations during the cold chain can accelerate quality deterioration by altering microbial activity, water status, and biochemical processes, with the extent of these effects closely linked to the structural and compositional characteristics of the food matrix.

However, unlike animal-derived foods, tofu is a typical high-moisture plant-protein gel system, and its quality changes are not only affected by microbial activity and oxidative reactions but are also closely related to the water-binding capacity of the protein gel network. The water in tofu is largely entrapped and immobilized within the three-dimensional gel network formed by soybean proteins, making its water retention and textural stability particularly susceptible to changes in storage conditions (10, 11). Therefore, maintaining gel stability while delaying microbial, physicochemical, and flavor deterioration has been a key focus of tofu storage research. Van Campenhout et al. (5) found that modified-atmosphere packaging delayed microbial growth in refrigerated tofu and improved its storage stability. More recently, Wu et al. (4) investigated fermented soybean whey-based tofu during refrigerated storage at 10 °C and found that nisin inhibited microbial proliferation and biogenic amine accumulation while helping maintain its water-holding capacity and flavor quality. However, these studies mainly focused on tofu quality preservation under relatively constant temperature conditions, and therefore do not fully reflect the effects of repeated temperature fluctuations encountered during actual cold-chain logistics. Under such dynamic temperature conditions, microbial growth and spoilage metabolism may be altered, accompanied by changes in water distribution, water-holding capacity, texture, and volatile flavor profiles. Although the effects of cold chain temperature fluctuations on quality changes of meat and aquatic products have been extensively reported, their impact on high-moisture plant-protein gel foods remains poorly understood. In particular, how the quality and flavor characteristics of pre-packaged tofu evolve in response to different degrees of temperature fluctuation has yet to be systematically investigated. Therefore, investigating the quality deterioration and flavor evolution of pre-packaged tofu under temperature fluctuation conditions is important for improving quality control in the cold-chain logistics of plant-protein foods.

Hence, this study simulated stable low-temperature storage and two temperature fluctuation conditions during cold-chain logistics to systematically evaluate their effects on the storage quality and flavor evolution of pre-packaged tofu. Total bacterial counts, psychrophilic bacterial counts, pH, total volatile basic nitrogen (TVB-N), thiobarbituric acid-reactive substances (TBARS), biogenic amines, water-holding capacity, and textural properties were determined to characterize microbial and physicochemical changes during storage. Low-field nuclear magnetic resonance (LF-NMR) and magnetic resonance imaging (MRI) were used to characterize changes in water status, while electronic nose, gas chromatography-ion mobility spectrometry (GC-IMS), and gas chromatography-mass spectrometry (GC-MS) analyses were performed to evaluate changes in volatile flavor profiles. This study aimed to characterize the quality deterioration and flavor evolution of pre-packaged tofu under different cold-chain temperature conditions and to explore the potential associations among microbial proliferation, physicochemical deterioration, water status, texture, and volatile flavor changes, thereby providing experimental evidence for quality control during cold-chain transportation of plant-protein foods.

## 2 Materials and methods

### 2.1 Materials

Non-GMO soybean (protein content 40%) was originally cultivated in Anhui Province, China and supplied by Jinzai Food Group Co., Ltd (Yueyang, China). Fermented soybean whey (FSW, total acid content: 4.49 ± 0.1 g/L, protease content: 9.51 ± 0.2 U/mL) was obtained from Hunan Junyifu food Co., Ltd. (Shaoyang, China) Methyl nonanoate (purity ≥ 99%) was purchased from Shanghai Maclin Biochemical Technology Co., Ltd. (Shanghai, China) Dan sulfonate chloride and biological amine analysis standard substances [High-Performance Liquid Chromatography (HPLC) grade ≥ 98%] were sourced from Shanghai yuanye Bio-Technology Co., Ltd. (Shanghai, China) All other chemical reagents were of analytical grade and purchased from Sinopharm Chemical Reagent Co., Ltd. (Shanghai, China)

### 2.2 Sample preparation

Fermented soybean whey-based tofu was produced following a method described by Huang et al. (12). The resulting tofu was cut into blocks (250 g; 7.0 × 10.0 × 4.0 cm), and aseptically packaged into sterile polyethylene containers, each supplemented with approximately 90 g of an immersion solution containing 0.2% chitosan oligosaccharides (pH 5.0, 2 mg/mL, w/w). The packages were heat-sealed (185 °C for 25 s) and subjected to reverse-pressure sterilization (85 °C for 30 min). The samples were randomly divided into three groups and stored under three simulated cold chain logistics conditions for 35 days, as illustrated in Figure 1. Group CK represents an ideal cold chain with constant temperature; Group T1 represents the supermarket logistics model, simulating frequent and severe temperature fluctuations and cold chain interruptions during distribution center transfers, store unloading, and shelf display; Group T2 represents the e-commerce logistics model, simulating the long-haul trunk transportation and terminal delivery process involving prolonged low-temperature storage accompanied by a single brief exposure to ambient temperature. In designing these programs, 4 °C was used to represent refrigerated transportation, 10 °C to represent refrigerated storage, and 20 °C to simulate short-term temperature exposure during operations such as loading, unloading, transfer, and terminal delivery. Different combinations and durations of these temperature stages were then used to reproduce the characteristic temperature-exposure patterns of the two logistics modes under controlled experimental conditions. Quality indicators were analyzed at predetermined intervals (0, 2, 5, 7, 10, 15, 20, 25, 30, 35 days) during storage. Three independently packaged tofu samples from the same production batch were used as biological replicates (*n* = 3) for each distinct temperature condition.

**Figure 1:**
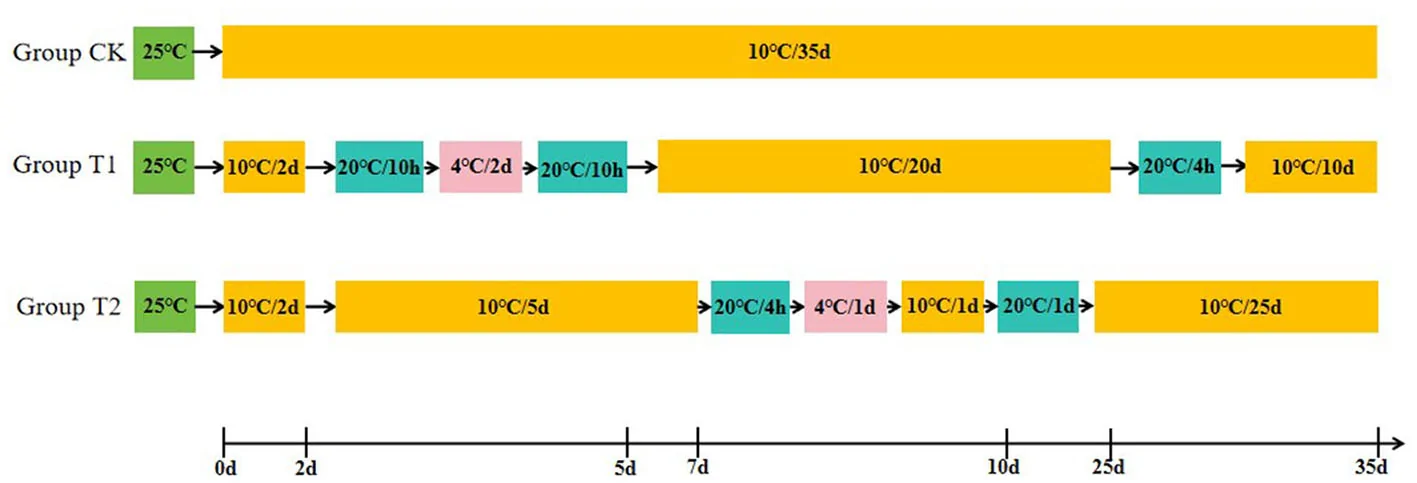
Schematic diagram of the simulated cold chain logistics conditions.

### 2.3 Microbiological analyses

A 25 g sample of tofu was homogenized with 225 mL of sterile saline (0.85%, w/v) in a sterile stomacher bag. The homogenate was then serially diluted, and appropriate dilutions were spread-plated onto plate count agar. The total viable count (TVC) and psychrotrophic bacterial counts (PBC) were enumerated after incubating at 36 ± 1 °C for 48 h and 7 ± 1 °C, respectively.

### 2.4 pH measurements

10.0 g tofu samples were homogenized with 90 mL of deionized water for 3 min, and pH values were determined using a digital pH meter (PHB-5, Inesa Scientific Instrument Co., Ltd, China).

### 2.5 Total volatile basic nitrogen (TVB-N)

According to the GB 5009.228-2016 national food safety standard, TVB-N content was determined using a Kjeldahl nitrogen tester (VAP450, Gerhardt, Konigswinter, German). TVB-N content was expressed as mg/100 g.

### 2.6 Thiobarbituric acid-reactive substances (TBARS)

The TBARS value was determined as described by Huang et al. (2). Namely, tofu samples (5.0 g) were homogenized with 50 mL of 20% (w/v) trichloroacetic acid mixture (containing 0.1% EDTA-Na_2_), followed by filtration to remove residues. Five milliliters the supernatant was mixed with 5 mL of thiobarbituric acid solution and incubated at 90 °C for 30 min. The absorbance was measured at 532 nm. The TBARS value was calculated using a standard curve prepared with various concentrations of malondialdehyde (MDA) standard solutions and expressed as mg of malondialdehyde equivalents per 100 g of sample (mg/100 g).

### 2.7 Moisture content

The moisture content was determined according to the GB 5009.3-2016 Chinese standard method. Tofu samples were accurately weighed (recorded as m_1_), placed in an oven at 105 °C, and dried to a constant weight (m_2_). The moisture content of tofu was calculated using the following equation:


$$\begin{array}{l} \text{Moisturecontent}\left(\text{\%}\right)=\left(m_{1}-m_{2}\right)/m_{1}\times 100\text{\%} \end{array}$$

### 2.8 Water-holding capacity (WHC)

The WHC was measured using a method by Chen et al. (13) with minor modifications. Briefly, 2 g of tofu was weighed (denoted as *m*_1_), wrapped in two layers of filter paper, and centrifuged at 6,000 rpm for 10 min at 15 °C. After blotting the residual surface water, the sample was reweighed and recorded as *m*_2_. The WHC (%) of the tofu was calculated as:


$$\begin{array}{l} \text{WHC}\left(\text{\%}\right)=\left(m_{2}/m_{1}\right)\times 100\text{\%} \end{array}$$

### 2.9 Biogenic amine analysis

The determination of biogenic amines was carried out according to a method reported by Zhang et al. (14) with minor modifications. Tofu samples (25 g) were homogenized with 20 mL of 0.4 mol/L perchloric acid solution and extracted ultrasonically for 20 min. After filtration, the filtrate was collected, and the residue was re-extracted with a perchloric acid solution. All extracts were combined and diluted to 50 mL with a perchloric acid solution. For derivatization, the extracted solution was reacted with dansyl chloride prior to chromatographic analysis. The derivatized samples were separated on a C18 column using ultrapure water and acetonitrile as mobile phases. The gradient elution procedure was applied according to the established method. The qualitative and quantitative determination of biogenic amines was conducted by comparing the retention times and peak areas with those of external standards. The concentrations of individual biogenic amines were calculated from the corresponding calibration curves.

### 2.10 Electronic nose analysis

The odor profiles of prepackaged tofu were analyzed using a portable electronic nose system (PEN3, Win Muster Airsense Analytics Inc., Schwerin, Germany). Approximately 4.00 g of chopped sample was accurately weighed and placed into a 30 mL standard headspace vial. The vial was sealed and equilibrated in a water bath at 50 °C for 6 min to allow volatile enrichment. The measurement parameters were set as follows: detection time of 100 s, cleaning time of 100 s, and a constant flow rate of 400 mL/min to pump the headspace gas into the sensor array. The measurements were conducted at room temperature, and sensor signals were recorded at 1 s intervals. Each sample was analyzed in triplicate to ensure reproducibility, and the sensor data was automatically processed using WinMuster software.

### 2.11 Texture tests

The textural parameters (hardness, springiness, chewiness, resilience) of prepackaged tofu were measured using a Texture Analyzer (TA1, Lloyd, Britain) using a three-point sampling method. The testing conditions were as follows: texture profile analysis (TPA) mode, P35 cylindrical flat probe, pre-test force 0.05 N, compression deformation 50%, compression speed 60 mm·min^−1^, post-test retract speed 50 mm·min^−1^, interval time 5 s.

### 2.12 LF-NMR and MRI

The sample was cut into small pieces (3.0 × 3.0 × 3.0 cm), wrapped in polyethylene cling film, and placed in an NMR tube. The Carr-Purcell-Meiboom-Gill (CPMG) sequence was used to collect the transverse relaxation time (T_2_) signal of the sample, and the imaging software was used to obtain a nuclear magnetic resonance image.

The CPMG parameters were set as follows: SF (MHz) = 21, RFD (ms) = 0.020, O1 (Hz) = 117,366.24, RG1 (db) = 20.0, P1 (us) = 13.50, DRG1 = 1, TD = 1,024, DR = 1, PRG = 0, TW (ms) = 3,000.00, NS = 8, P2 (us) = 27.04, TE (ms) = 0.300, NECH = 5,000.

The MRI parameters were set as follows: Average = 4, Slice Count = 5, Slice Thickness (mm) = 5.50, Slice Gap (mm) = 1.00, FOV = 100, Waiting time (TR) = 500 ms, Echo time = 20 ms, Phase encoding steps = 192, Read size = 256.

### 2.13 GC-IMS volatile flavor compound analysis

Volatile compounds were analyzed using a GC-IMS system (FlavourSpec 1 H1-100053, GAS, Germany) equipped with an autosampler (Combi PAL, CTC, Switzerland) and an MXT-5 column (15 m × 0.53 mm, 1.0 μm, Restek, Bellefonte, USA). Approximately 2 g of tofu sample was placed in a 15 mL headspace vial, incubated at 60 °C for 10 min with shaking at 500 r/min, and 500 μL of headspace gas was injected at 80 °C. GC oven temperature was maintained at 60 °C. High-purity nitrogen (≥99.999%) was used as carrier gas at a programmed flow rate: 2 mL/min for 0–2 min, ramped to 100 mL/min over 2–25 min, and held at 100 mL/min for 25–30 min. The IMS drift tube (530 mm) was operated at 45 °C with a drift gas flow of 150 mL/min. Compounds were identified using C4-C9 n-ketone standards and the built-in IMS database based on retention indices and drift times.

### 2.14 HS-GC-MS volatile flavor compound analysis

A method reported by Wu et al. (4) was used with slight modifications. Briefly, tofu samples (3 g) were placed into a 30 mL headspace vial, and 1 μL of methyl nonanoate solution (2.75 mg/mL, dissolved in methanol) was added as an internal standard. Volatile compounds were extracted using a 50/30 μm DVB/CAR/PDMS fiber (1 cm, Bellefonte, USA) at 80 °C for 35 min. After extraction, the fiber was inserted into the GC injection port and desorbed at 250 °C for 5 min. Gas chromatography analysis was performed using a DB-5MS capillary column (30 m × 0.25 mm × 0.25 μm). Helium (purity ≥99.999%) was used as the carrier gas at a flow rate of 1.0 mL/min. The oven temperature was programmed as follows: the initial temperature was maintained at 40 °C for 3 min, increased to 100 °C at 4 °C/min and held for 2 min, then increased to 230 °C at 8 °C/min and maintained for 5 min. Mass spectrometry was performed under electron ionization (EI) mode at 70 eV, with a scanning range of 33–550 m/z. The volatile compounds were identified by matching the mass spectra with the National Institute of Standards and Technology (NIST) library, with a match similarity threshold of >80%. The volatile compounds were semi-quantified using methyl nonanoate as the internal standard. The semi-quantitative content of each volatile compound was calculated from the ratio of its peak area to that of the internal standard, the amount of internal standard added, and the sample mass, and expressed as μg/kg.

### 2.15 Statistical analysis

All experiments were performed in triplicate, and the results were expressed as mean ± standard deviation (SD). Two-way analysis of variance (ANOVA) was performed using IBM SPSS Statistics 26 (IBM Corp., Armonk, NY, USA) to evaluate the effects of storage time, cold-chain treatment, and their interaction. Significant differences between means were determined by Duncan's multiple range test at a significance level of *P* < 0.05. Data visualization was conducted using Origin 2024 (OriginLab Corp., Northampton, MA, USA), and orthogonal partial least squares discriminant analysis (OPLS-DA) was performed using SIMCA-P 14.1 (Umetrics, Umea, Sweden).

## 3 Results and discussion

### 3.1 Microbial proliferation

Microbial proliferation is an important factor associated with the quality deterioration and shelf-life shortening of refrigerated soy products, and therefore clarifying the change pattern of microbial populations under temperature fluctuation conditions is of great significance for understanding food deterioration during cold-chain storage. As shown in Table 1, the TVC and PBC of pre-packaged tofu under different logistics modes both showed a continuous increasing trend with storage time. At the initial stage (0–2 d), the microbial counts of all groups were at low levels, indicating that low-temperature environments effectively inhibited rapid microbial growth. However, with the extension of storage time, significant differences in microbial growth rate occurred under different temperature conditions. Compared with the constant-temperature cold chain group (CK), the temperature fluctuation treatment groups (T1 and T2) showed more rapid increases in TVC (*P* < 0.05). Among these, the T1 group exhibited the most notable promoting effect, with its TVC rapidly increasing from 2.76 lg CFU/g at 10 d to 6.52 lg CFU/g at 35 d, significantly higher than that of the CK group (5.24 lg CFU/g) and T2 group (6.33 lg CFU/g) (*P* < 0.05). The change trend of PBC was consistent with that of TVC. At 35 d, PBC of the T1 group reached 3.24 lg CFU/g, always higher than that of the CK (2.41 lg CFU/g) and T2 groups (3.17 lg CFU/g), indicating that larger temperature fluctuations were associated with greater proliferation of psychrotrophic bacteria. Notably, the microbial counts of all treatment groups showed an obvious accelerated growth trend after 10 d, suggesting that the middle and late stages of storage were critical periods for microbial proliferation under the tested cold-chain conditions (15). Repeated warming during temperature fluctuations may create transient conditions favorable for microbial growth and metabolic activity, thereby weakening the inhibitory effect that stable low-temperature conditions have on microbial growth (16). This result was consistent with the findings of Chen et al. (8) who showed that temperature fluctuations promoted microbial growth during the cold chain storage of large yellow croaker, supporting the importance of temperature stability for controlling microbial proliferation in refrigerated foods. In summary, temperature fluctuation treatments were associated with significantly greater microbial proliferation in pre-packaged tofu, with the T1 treatment generally showing the highest microbial counts. The concurrent increase in microbial counts provided a basis for further examining their associations with subsequent changes in spoilage-related physicochemical indicators and volatile profiles.

**Table 1:** Changes in microorganism of prepackaged tofu under different logistics modes.

Indexes	Sample	Storage time/d									
		0	2	5	7	10	15	20	25	30	35
TVC Lg (CFU/g)	CK	<1	0.45 ± 0.13^Ia^	0.92 ± 0.07^Hc^	1.23 ± 0.11^Gb^	2.19 ± 0.15^Fc^	3.11 ± 0.07^Eb^	3.52 ± 0.24^Dc^	4.13 ± 0.22^Cc^	4.63 ± 0.06^Bc^	5.24 ± 0.11^Ac^
	T1	<1	0.41 ± 0.11^Ia^	1.11 ± 0.12^Ha^	1.44 ± 0.17^Ga^	2.76 ± 0.08^Fb^	3.32 ± 0.05^Ea^	3.88 ± 0.11^Da^	4.74 ± 0.13^Ca^	4.95 ± 0.24^Ba^	6.52 ± 0.07^Aa^
	T2	<1	0.46 ± 0.08^Ia^	1.03 ± 0.14^Hb^	1.24 ± 0.21^Gb^	2.83 ± 0.24^Fa^	3.27 ± 0.12^Eb^	3.65 ± 0.21^Db^	4.66 ± 0.15^Cb^	4.88 ± 0.12^Bb^	6.33 ± 0.16^Ab^
PBC Lg (CFU/g)	CK	<1	<1	0.22 ± 0.04^Hb^	0.43 ± 0.10^Gc^	0.59 ± 0.13^Fb^	0.78 ± 0.06^Eb^	1.01 ± 0.03^Dc^	1.37 ± 0.13^Cb^	1.85 ± 0.02^Bc^	2.41 ± 0.05^Ac^
	T1	<1	<1	0.48 ± 0.05^Ha^	0.52 ± 0.08^Ga^	0.66 ± 0.11^Fa^	0.91 ± 0.04^Ea^	1.13 ± 0.06^Da^	1.51 ± 0.09^Ca^	2.12 ± 0.10^Ba^	3.24 ± 0.06^Aa^
	T2	<1	<1	0.20 ± 0.07^Hb^	0.47 ± 0.3^Gb^	0.60 ± 0.09^Fb^	0.93 ± 0.07^Ea^	1.09 ± 0.11^Db^	1.50 ± 0.07^Ca^	2.07 ± 0.13^Bb^	3.17 ± 0.06^Ab^

Different uppercase letters indicate significant differences among storage times within the same sample (*P* < 0.05). Different lowercase letters indicate significant differences among samples (CK, T1, and T2) at the same storage time (*P* < 0.05). Means sharing the same letter are not significantly different.

### 3.2 Physicochemical deterioration

#### 3.2.1 pH

Changes in pH can reflect changes in the physicochemical environment associated with food spoilage and thus serve as an important indicator to evaluate the spoilage progression of products. As shown in Figure 2A, the pH values of pre-packaged tofu in all groups showed a trend of initially decreasing and then increasing, reflecting a stage-dependent change in the physicochemical environment during storage. The initial pH of all samples was 6.22. With the extension of storage time, pH continuously decreased from 0 to 10 d. Among these, the CK, T1 and T2 groups reached their minimum values of 5.75, 5.62, and 5.67 at 10 d, respectively. Moreover, the pH of the T1 group was significantly lower than that of the CK group (*P* < 0.05), indicating that temperature fluctuations were associated with a more pronounced pH decrease, which may be related to enhanced microbial activity and the accumulation of acidic metabolites (2). Notably, after 10 d, the pH gradually increased in all groups, but the recovery rates differed significantly among treatment groups. Compared with the CK group, temperature fluctuation treatment groups showed a faster pH increase trend, with the T1 group exhibiting the most notable change. At 35 d, the pH of the CK, T1, and T2 groups reached 6.27, 6.53, and 6.44, respectively, with the pH of the T1 group being significantly higher than that of the CK and T2 groups (*P* < 0.05). A later increase in pH is usually associated with the accumulation of alkaline nitrogen-containing compounds (e.g., volatile amines) produced by spoilage microbial metabolism (17). Combined with the TVC and PBC results described above, the more pronounced pH increase under temperature fluctuation conditions coincided with greater microbial proliferation and the accumulation of spoilage-related nitrogenous compounds, as further reflected by the TVB-N and biogenic amine results. Similarly, Lan et al. (18) found that temperature fluctuations during cold chain transportation could induce decreasing pH trends followed by increases in aquatic products, consistent with the results of this study. In summary, temperature fluctuation treatments were associated with greater microbial proliferation and more pronounced pH changes in pre-packaged tofu, consistent with more extensive spoilage during storage.

**Figure 2:**
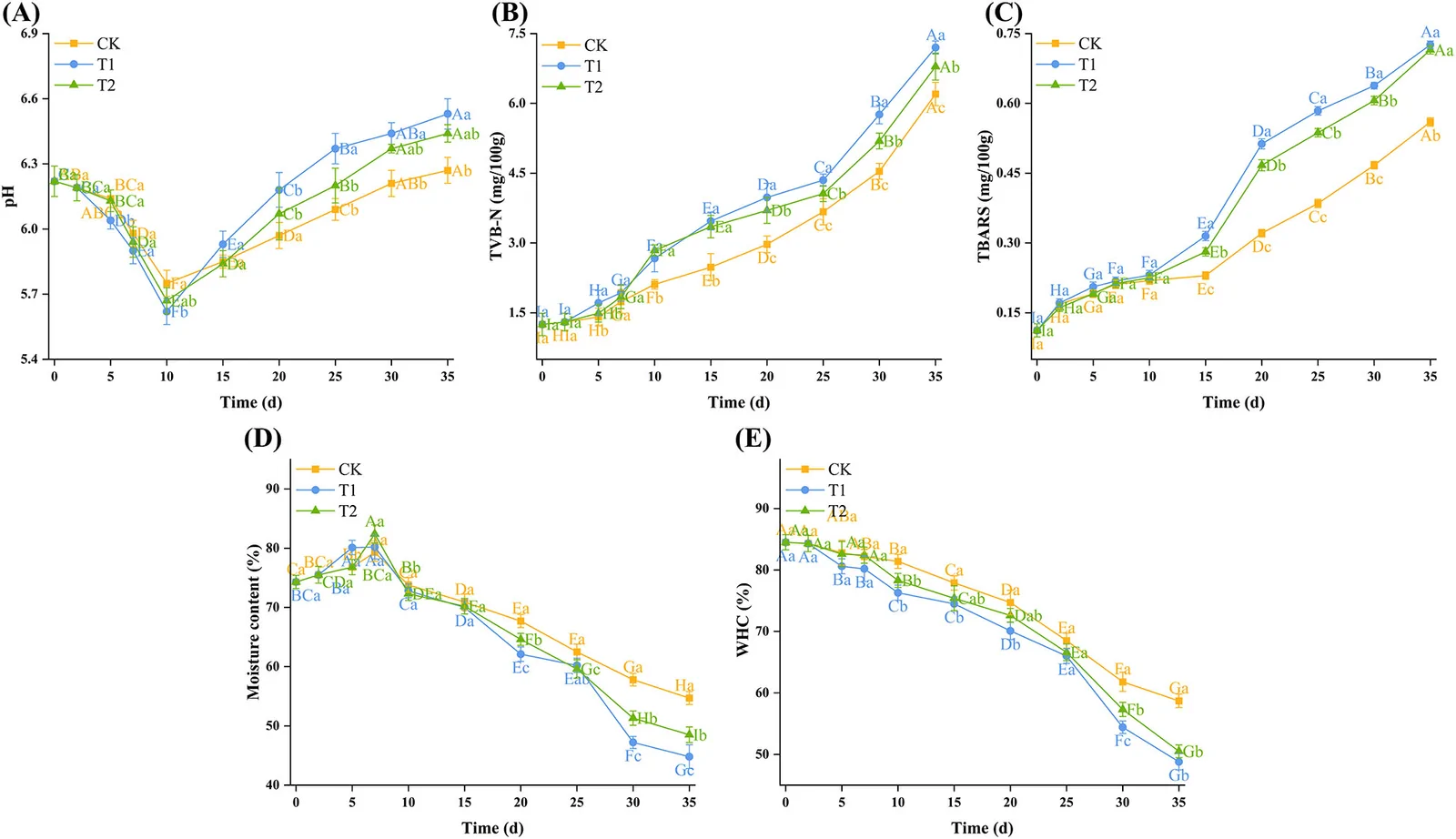
Changes in physicochemical properties of prepackaged tofu under different cold chain logistics conditions. **(A)** pH; **(B)** total volatile basic nitrogen (TVB-N); **(C)** thiobarbituric acid-reactive substances (TBARS); **(D)** moisture content; **(E)** water holding capacity (WHC). Different uppercase letters indicate significant differences among storage times within the same sample (*P* < 0.05). Different lowercase letters indicate significant differences among samples (CK, T1, and T2) at the same storage time (*P* < 0.05). Means sharing the same letter are not significantly different.

#### 3.2.2 TVB-N

TVB-N content is a key indicator for evaluating the accumulation of volatile basic nitrogenous compounds and the degree of food spoilage during storage. As shown in Figure 2B, the TVB-N contents of pre-packaged tofu under different logistics modes showed a continuous increasing trend with storage time. The initial TVB-N content of all samples was 1.25 mg/100 g. At an early storage stage (0–10 d), TVB-N increased slowly in all treatment groups, although the T1 and T2 groups exhibited higher accumulation levels. At 10 d, TVB-N of the CK, T1, and T2 groups reached 2.11, 2.67, and 2.84 mg/100 g, respectively, with the TVB-N of the T1 and T2 groups being significantly higher than that of the CK group (*P* < 0.05). This result indicated that temperature fluctuations were associated with enhanced accumulation of nitrogenous metabolites, accompanied by increased microbial activity. With further extension of storage time, TVB-N content in all groups entered a rapid growth stage, and the differences among treatments gradually increased. At 35 d, TVB-N of the T1 group reached 7.20 mg/100 g, significantly higher than that of the T2 group (6.79 mg/100 g) and the CK group (6.20 mg/100 g) (*P* < 0.05), indicating the greatest accumulation of TVB-N in the T1 group by the end of storage. Combined with the microbial results described above, this greater TVB-N accumulation was associated with higher microbial counts. Previous studies have linked microbial activity during food spoilage to protein degradation and the subsequent formation of volatile amines and other alkaline nitrogenous compounds (19); however, proteolytic enzyme activity was not directly measured in the present study. Therefore, changes in TVB-N further indicated an accelerated spoilage process in pre-packaged tofu under temperature fluctuation conditions. This result was consistent with the findings of Lan et al. (18) who showed that cold chain temperature fluctuations promote protein degradation, further supporting the observed deterioration trend.

#### 3.2.3 TBARS

TBARS values are an important indicator for evaluating the degree of lipid oxidation, and its changes can reflect the accumulation level of secondary oxidation products such as malondialdehyde during the oxidation of unsaturated fatty acids. As shown in Figure 2C, the TBARS contents of pre-packaged tofu under different logistics modes continuously increased with storage time. The initial TBARS value of all samples was 0.112 mg/100 g. At the early storage stage (0–10 d), TBARS values increased slowly in all groups, and the differences among treatments were small, indicating that low-temperature environments effectively inhibited lipid oxidation. However, with the extension of storage time, TBARS values increased more markedly in the temperature fluctuation groups. After 15 d, the differences among different treatment groups gradually increased, among which the T1 group showed the fastest increase rate of TBARS, reaching 0.315 mg/100 g at 15 d, significantly higher than that of the CK group (0.230 mg/100 g) and the T2 group (0.281 mg/100 g) (*P* < 0.05). After further extension of storage time, TBARS values in all groups rapidly increased. At 35 d, TBARS of the T1 and T2 groups reached 0.726 and 0.715 mg/100 g, respectively, both significantly higher than that of the CK group (0.560 mg/100 g) (*P* < 0.05). The results indicated that temperature fluctuations were associated with greater lipid oxidation, as reflected by higher TBARS values, with larger fluctuations generally corresponding to greater TBARS increases. This greater increase may be associated with changes in the physicochemical environment during repeated warming, which could increase the susceptibility of lipids to oxidation (20–22). However, the specific pathways responsible for the enhanced oxidation were not directly investigated in the present study. The trend in TBARS values was consistent with the aforementioned microbial growth and TVB-N accumulation patterns, suggesting that microbial proliferation and lipid oxidation occurred concurrently during the accelerated deterioration of tofu under temperature fluctuation conditions.

#### 3.2.4 Moisture content and WHC

Water status is a key factor affecting the texture properties and storage stability of tofu, and its changes are closely related to the protein gel network structure and water migration. As shown in Figures 2D, E, the moisture content and WHC of pre-packaged tofu under different logistics modes both showed an overall decreasing trend with storage time, although some fluctuations occurred during the early storage stage. The initial moisture content and WHC of samples were 74.3% and 84.5%, respectively. During 0–7 d, the moisture content of all groups showed a temporary increase, with the T2 group reaching 82.4% at 7 d, which might be related to the redistribution of water between the tofu matrix and the surrounding packaging environment during the initial storage stage. With further extension of storage time, the moisture content and WHC of all groups continuously decreased, and the decreases in temperature fluctuation groups were significantly greater than those in the CK group. At 35 d, the moisture contents of the CK, T1 and T2 groups decreased to 54.7%, 44.8%, and 48.5%, respectively, with the T1 group being significantly lower than the CK and T2 groups (*P* < 0.05). Meanwhile, the WHC of the three groups decreased to 58.7%, 48.8%, and 50.5%, respectively, and temperature fluctuation treatments significantly weakened the water-holding capacity of tofu (*P* < 0.05). The WHC of tofu essentially depends on the compactness and integrity of its protein gel network (23). Wang et al. (24) found in a cold chain study on pork that temperature fluctuations led to a decrease in WHC, consistent with the results of this study. In summary, constant-temperature cold chain conditions were conducive to reducing water loss and maintaining the WHC of tofu, thereby better preserving product quality. This may be associated with differences in the stability of the tofu gel matrix across temperature conditions. This result also provided the basis for subsequent LF-NMR and MRI analyses of changes in internal water status of tofu under different logistics modes.

### 3.3 Biogenic amines

Biogenic amines are important nitrogenous compounds that can accumulate during food storage, and their formation is commonly associated with microbial amino acid decarboxylation (25). As shown in Table 2, with the extension of storage time, spoilage-related biogenic amines (tryptamine, phenethylamine, putrescine, cadaverine, histamine, tyramine) in pre-packaged tofu showed a continuous accumulation trend, while endogenous polyamines (spermidine, spermine) gradually decreased, indicating marked changes in the biogenic amine profile during cold chain storage. The biogenic amine contents in all groups of fresh samples were basically consistent, with putrescine, spermidine, and spermine being the main components. With the extension of storage time, spoilage-related biogenic amines including tryptamine, phenethylamine, putrescine, cadaverine, histamine, and tyramine gradually increased, and the accumulation rates in temperature fluctuation treatment groups were significantly higher than those in the constant-temperature group. Especially at the late storage stage (25–35 d), the biogenic amine contents in all groups increased rapidly, which coincided with the marked increase in microbial counts, suggesting an association between microbial proliferation and biogenic amine accumulation. Among these, at 35 d, the contents of tryptamine, phenethylamine, putrescine, cadaverine, histamine, and tyramine in the T1 group reached 3.10, 2.02, 2.62, 1.52, 1.93, and 1.12 mg/100 g, respectively, all higher than those in the CK and T2 groups, consistent with greater biogenic amine accumulation under larger temperature fluctuations. The changes in different types of biogenic amines further reflected changes in spoilage-related amine profiles during the storage of pre-packaged tofu. Putrescine and cadaverine mainly originate from decarboxylation of ornithine and lysine, serving as important markers of enhanced spoilage microbial activity; histamine and tyramine are associated with decarboxylation of histidine and tyrosine, and their continuous accumulation may be associated with the availability and microbial decarboxylation of their corresponding amino acid precursors (25). In contrast, the contents of spermidine and spermine gradually decreased with storage time, which might be related to polyamine degradation and changes in microbial activity during storage (26). Combined with the results of microbial counts and TVB-N changes described above, temperature fluctuations promoted microbial proliferation and accelerated biogenic amine accumulation, while the concurrent changes in these indicators suggest an association between microbial activity and amine formation. Among these, the T1 group exhibited the highest biogenic amine levels, consistent with its higher TVC and TVB-N contents, supporting the association between enhanced microbial activity and the accelerated spoilage of pre-packaged tofu under temperature fluctuation conditions. The accumulation of biogenic amines therefore provided an additional indicator of spoilage progression and complemented the subsequent analysis of volatile flavor changes.

**Table 2:** Changes in biogenic amine contents of prepackaged tofu under different cold chain logistics conditions.

Biogenic amines	Sample	Storage time (d)					
		0	5	10	25	30	35
Tryptamine	CK	0.7608 ± 0.011^Fa^	0.8151 ± 0.010^Eb^	0.8808 ± 0.006^Dc^	1.5471 ± 0.020^Cc^	1.9166 ± 0.022^Bc^	2.2053 ± 0.007^Ac^
	T1	0.7608 ± 0.011^Fa^	0.8433 ± 0.009^Ea^	0.9311 ± 0.021^Da^	1.9187 ± 0.004^Ca^	2.3331 ± 0.020^Ba^	3.1007 ± 0.010^Aa^
	T2	0.7608 ± 0.011^Fa^	0.8166 ± 0.015^Eb^	0.9032 ± 0.013^Db^	1.8733 ± 0.016^Cb^	2.1056 ± 0.015^Bb^	2.9476 ± 0.021^Ab^
Phenylethylamine	CK	0.0985 ± 0.002^Fa^	0.0998 ± 0.013^Eb^	0.1277 ± 0.010^Dc^	0.8816 ± 0.023^Cc^	1.1071 ± 0.019^Bc^	1.4000 ± 0.030^Ac^
	T1	0.0985 ± 0.002^Fa^	0.1211 ± 0.008^Ea^	0.1617 ± 0.021^Da^	1.1134 ± 0.010^Ca^	1.5363 ± 0.025^Ba^	2.1105 ± 0.023^Aa^
	T2	0.0985 ± 0.002^Fa^	0.0993 ± 0.012^Eb^	0.1410 ± 0.006^Db^	1.1055 ± 0.015^Cb^	1.4117 ± 0.007^Bb^	2.0212 ± 0.015^Ab^
Putrescine	CK	0.5203 ± 0.010^Fa^	0.5441 ± 0.010^Ec^	0.7567 ± 0.003^Dc^	1.5052 ± 0.022^Cc^	1.8434 ± 0.016^Bc^	2.0703 ± 0.007^Ac^
	T1	0.5203 ± 0.010^Fa^	0.7123 ± 0.009^Ea^	0.9171 ± 0.011^Da^	1.8673 ± 0.017^Ca^	2.1101 ± 0.011^Ba^	2.6202 ± 0.011^Aa^
	T2	0.5203 ± 0.010^Fa^	0.5511 ± 0.005^Eb^	0.8807 ± 0.015^Db^	1.7752 ± 0.009^Cb^	2.0477 ± 0.019^bB^	2.5383 ± 0.010^Ab^
Cadaverine	CK	0.1164 ± 0.011^Fa^	0.1347 ± 0.013^Ec^	0.1721 ± 0.022^Dc^	0.6224 ± 0.020^Cc^	0.8779 ± 0.017^Bc^	1.1768 ± 0.010^Ac^
	T1	0.1164 ± 0.011^Fa^	0.1552 ± 0.010^Ea^	0.2101 ± 0.013^Da^	0.8336 ± 0.033^Ca^	1.1102 ± 0.025^Ba^	1.5232 ± 0.021^Aa^
	T2	0.1164 ± 0.011^Fa^	0.1411 ± 0.007^Eb^	0.1977 ± 0.022^Db^	0.8170 ± 0.018^Cb^	1.0867 ± 0.006^Bb^	1.5170 ± 0.023^Ab^
Histamine	CK	0.1066 ± 0.010^Fa^	0.1082 ± 0.006^Ec^	0.2222 ± 0.010^Dc^	0.8651 ± 0.000^Cc^	1.1293 ± 0.010^Bc^	1.4257 ± 0.012^Ac^
	T1	0.1066 ± 0.010^Fa^	0.1111 ± 0.004^Ea^	0.5354 ± 0.008^Da^	1.3192 ± 0.011^Ca^	1.6116 ± 0.015^Ba^	1.9274 ± 0.009^Aa^
	T2	0.1066 ± 0.010^Fa^	0.1094 ± 0.011^Eb^	0.4025 ± 0.014^Db^	1.2325 ± 0.011^Cb^	1.6004 ± 0.019^bB^	1.8413 ± 0.011^Ab^
Tyramine	CK	0.0288 ± 0.001^Fa^	0.0621 ± 0.003^Ec^	0.0809 ± 0.006^Dc^	0.4064 ± 0.007^Cc^	0.6353 ± 0.005^Bc^	0.8927 ± 0.012^Ac^
	T1	0.0288 ± 0.001^Fa^	0.0736 ± 0.000^Ea^	0.1522 ± 0.005^Da^	0.6676 ± 0.005^Ca^	0.8472 ± 0.010^Ba^	1.1220 ± 0.008^Aa^
	T2	0.0288 ± 0.001^Fa^	0.0656 ± 0.005^Eb^	0.1377 ± 0.003^Db^	0.6512 ± 0.010^Cb^	0.8235 ± 0.011^Bb^	1.0827 ± 0.007^Ab^
Spermidine	CK	1.5385 ± 0.003^Aa^	1.5300 ± 0.010^Ba^	1.5214 ± 0.012^Ca^	1.3509 ± 0.008^Da^	1.3232 ± 0.011^Ea^	1.1538 ± 0.010^Fa^
	T1	1.5385 ± 0.003^Aa^	1.5227 ± 0.011^Bb^	1.5006 ± 0.011^Cc^	1.2345 ± 0.012^Dc^	1.1076 ± 0.010^Eb^	1.0703 ± 0.012^Fc^
	T2	1.5385 ± 0.003^Aa^	1.5300 ± 0.007^Ba^	1.5133 ± 0.015^Cb^	1.2867 ± 0.010^Db^	1.2217 ± 0.006^Eb^	1.1015 ± 0.011^Fb^
Spermine	CK	0.6745 ± 0.006^Aa^	0.6601 ± 0.002^Ba^	0.6338 ± 0.011^Ca^	0.5882 ± 0.006^Da^	0.5372 ± 0.005^Ea^	0.5104 ± 0.010^Fa^
	T1	0.6745 ± 0.006^Aa^	0.6357 ± 0.001^Bc^	0.6083 ± 0.010^Cc^	0.5208 ± 0.010^Dc^	0.5079 ± 0.006^Ec^	0.4738 ± 0.008^Fc^
	T2	0.6745 ± 0.006^Aa^	0.6566 ± 0.004^Bb^	0.6145 ± 0.005^Cb^	0.5387 ± 0.012^Db^	0.5185 ± 0.010^Eb^	0.4985 ± 0.011^Fb^

Different uppercase letters indicate significant differences among storage times within the same sample (*P* < 0.05). Different lowercase letters indicate significant differences among samples (CK, T1, and T2) at the same storage time (*P* < 0.05). Means sharing the same letter are not significantly different.

### 3.4 Textural properties

Texture properties are important indicators for evaluating the structural integrity and textural quality changes of tofu. As shown in Figure 3, with the extension of storage time, the hardness, springiness, chewiness, and resilience of all samples showed decreasing trends, but the deterioration rates under different logistics modes exhibited notable differences. Fresh tofu mainly relies on the three-dimensional gel network formed by soybean proteins to maintain structural stability (1), and previous studies have associated microbial activity and protein degradation during storage with weakening of protein interactions and deterioration of gel properties (27). As such, hardness serves as an important indicator of the mechanical strength of the tofu gel. As shown in Figure 3A, the hardness of all samples gradually decreased with storage time, and at 35 d, the hardness of the CK, T2 and T1 groups decreased from an initial value of 2.31 N to 1.74, 1.51, and 1.44 N, respectively. Among these, the T1 group showed the largest decrease in hardness, indicating that larger temperature fluctuations were associated with greater loss of gel mechanical strength, while stable cold chain conditions effectively delayed the hardness decline of tofu. Notably, hardness changed relatively gradually in all groups from 15 to 35 d, whereas more pronounced changes were observed in the other textural parameters. This may be related to the distinct mechanical properties represented by these parameters: hardness mainly reflects resistance to compressive deformation, whereas springiness and resilience are more closely associated with recovery after deformation, and chewiness is influenced by multiple textural parameters. As storage progressed, the recovery ability of samples after resisting external forces gradually weakened, manifested as continuous decreases in springiness and resilience (Figures 3C, D). At 35 d of storage, the springiness of the CK, T2 and T1 groups decreased to 0.55, 0.46, and 0.43, respectively, and the resilience decreased to 0.26, 0.23, and 0.15, respectively, with the T1 group showing the most apparent decline (*P* < 0.05). Temperature fluctuations may alter the stability of the gel matrix and water distribution, which could contribute to the observed decrease in elastic recovery properties (28, 29). This result was consistent with the decreasing trend of WHC described above, suggesting that the loss of WHC was closely associated with texture deterioration. Chewiness is a composite TPA parameter influenced by hardness, cohesiveness, and springiness. At 35 d, the chewiness of the CK, T2, and T1 groups decreased to 93.33, 77.88, and 66.79, respectively, with the T1 group showing the most significant decline, indicating that temperature fluctuations further reduced overall textural performance of tofu. Notably, the decline rate of chewiness in the T1 group accelerated after 10 d, coinciding with the periods of rapid increase in TVB-N and TBARS, indicating that textural deterioration occurred concurrently with the accumulation of spoilage-related nitrogenous compounds and lipid oxidation products. In summary, temperature fluctuations accelerated the deterioration of tofu textural properties, as evidenced by decreases in hardness, springiness, chewiness, and resilience. This occurred concurrently with microbial proliferation, increased spoilage-related indicators, and reduced WHC, suggesting that alterations in the gel-water system may contribute to texture deterioration. Among these, the decrease in resilience suggested a progressive loss of the deformation-recovery ability of the gel structure (30).

**Figure 3:**
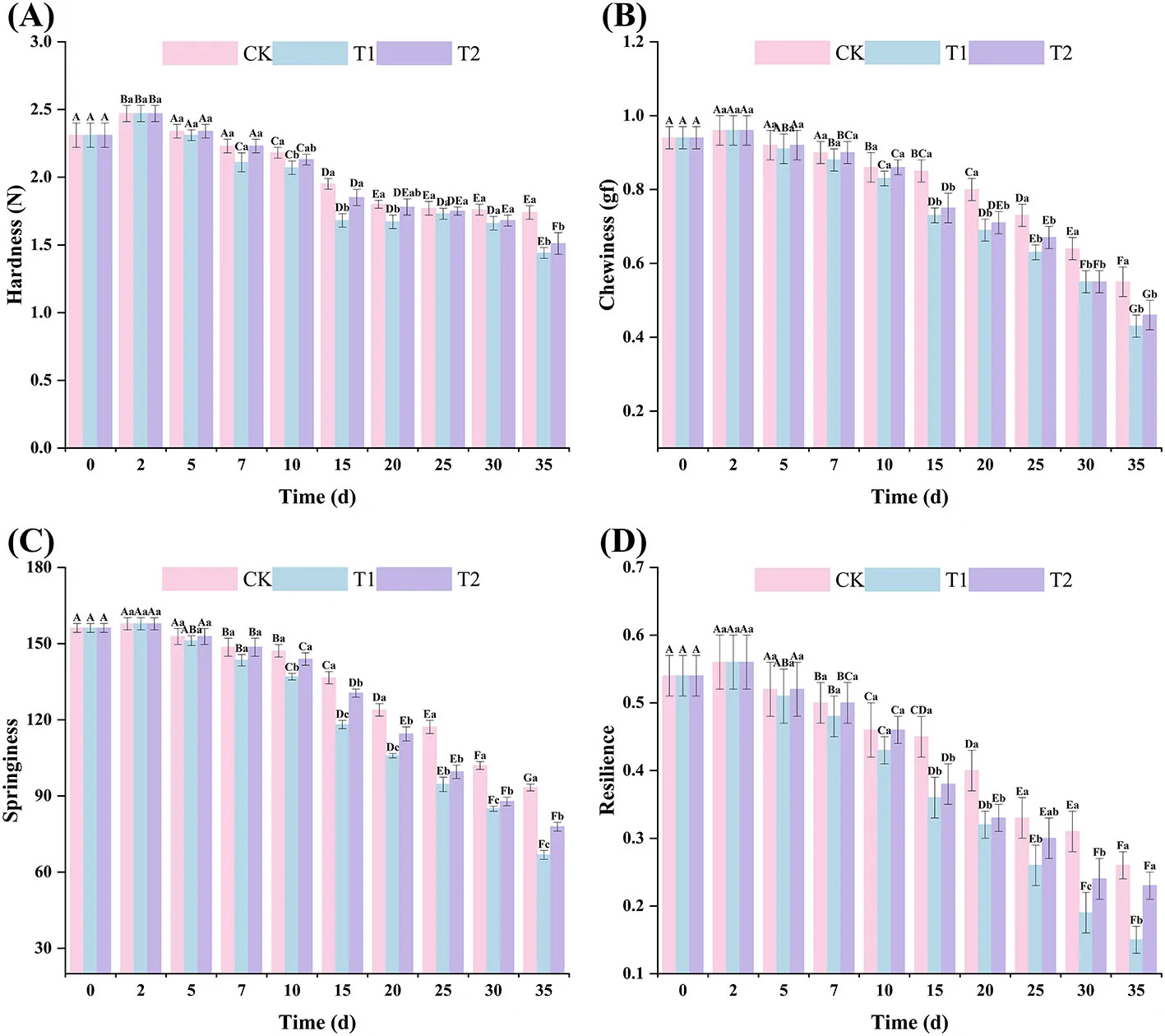
Changes in textural properties of prepackaged tofu under different cold chain logistics conditions. **(A)** hardness; **(B)** chewiness; **(C)** springiness; **(D)** resilience. Different uppercase letters indicate significant differences among storage times within the same sample (*P* < 0.05). Different lowercase letters indicate significant differences among samples (CK, T1, and T2) at the same storage time (*P* < 0.05). Means sharing the same letter are not significantly different.

### 3.5 LF-NMR

LF-NMR can characterize changes in water distribution and binding environments in food systems through shifts in transverse relaxation time (T_2_) and is an important method for analyzing water distribution and water-binding characteristics in gel-type foods. As shown in Figures 4A–C, the T_2_ relaxation spectra of pre-packaged tofu under different logistics conditions showed evident changes, indicating shifts in the distribution and relaxation characteristics of water populations within the tofu matrix during storage. According to the distribution of T_2_ relaxation time, the two major water populations were assigned to immobilized water and free water, with A_21_ and A_22_ representing their respective peak-area proportions. During storage, the T_2_ peak positions and peak intensities of all treatment groups changed to different degrees, among which the temperature fluctuation treatment groups showed more obvious changes. Compared with the CK group, the T1 and T2 groups showed more significant changes in T_2_ peak area and peak shape during storage, indicating that temperature fluctuations were associated with greater shifts in water distribution and binding environments inside tofu. The T_2_ spectra of the CK group changed relatively slowly throughout the storage period, suggesting that the water distribution remained relatively stable under constant-temperature cold-chain conditions, whereas the temperature fluctuation treatments showed more pronounced shifts in the T_2_ spectra, reflecting greater alterations in the water-binding environment of the tofu matrix.

**Figure 4:**
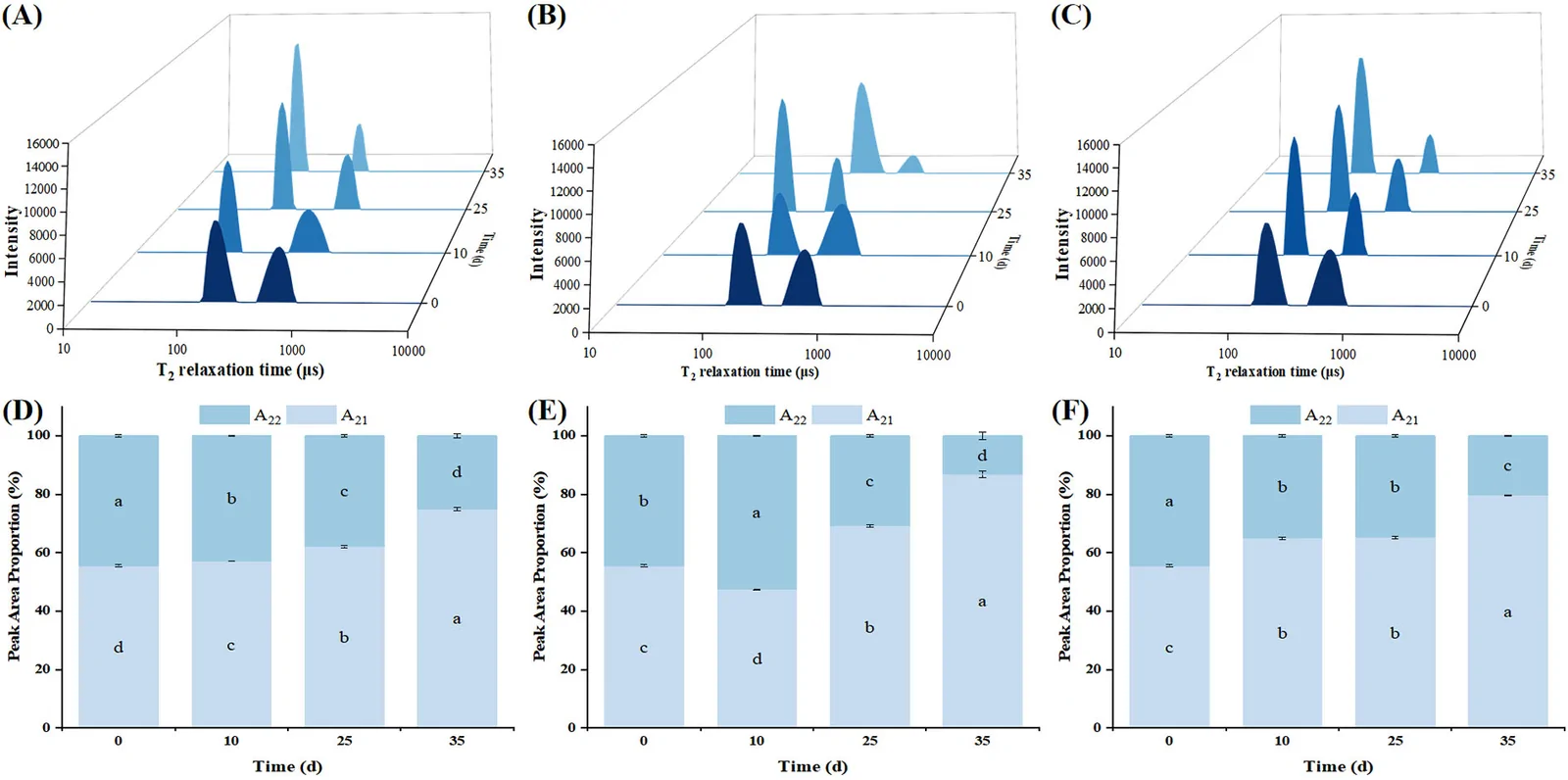
Water distribution in prepackaged tofu under different cold chain logistics conditions. **(A–C)** T2 relaxation spectra of CK, T1, and T2 groups; **(D–F)** corresponding proportions of A_21_ (immobilized water) and A_22_ (free water) peak areas. Different lowercase letters indicate significant differences among storage times within the same component (A_21_ or A_22_) (*P* < 0.05). Means sharing the same letter are not significantly different.

Further analysis of the changes in peak area ratios (Figures 4D–F) revealed that the ratios of A_21_ and A_22_ in all treatment groups changed significantly with storage time. The A_21_ ratio of the CK group gradually increased from 55.52% at 0 d to 75.03% at 35 d, while the A_22_ ratio decreased from 44.48% to 24.97%, indicating a progressive redistribution of the relative signal contributions of the two water populations under stable cold-chain conditions. In contrast, the temperature fluctuation groups showed more obvious time-dependent changes in water distribution, with the T1 group exhibiting the largest shifts. The A_21_ ratio of the T1 group decreased to 47.44% at 10 d, while the A_22_ ratio increased to 52.56%, indicating a transient shift in the relative distribution of water populations during early temperature fluctuations; subsequently, with the extension of storage time, the A_21_ ratio continuously increased, reaching 86.87% at 35 d, while the A_22_ ratio decreased to 13.13%, indicating continued redistribution of water populations during prolonged storage. The changing trend of the T2 group was similar to that of the T1 group, but the overall degree of change was lower, with the A_21_ and A_22_ ratios reaching 79.63% and 20.37% at 35 d, respectively. The above results indicated that temperature fluctuations significantly changed the distribution of different water populations inside tofu in a time-dependent manner. Tofu is mainly composed of a gel network formed by soybean proteins, and its water status is jointly regulated by protein-water interactions and the physicochemical environment of the gel matrix (31). Therefore, the variations in A_21_ and A_22_ observed in this study suggest a complex redistribution of water populations during storage rather than a simple unidirectional conversion between immobilized and free water. Such redistribution may be associated with changes in protein-water interactions and the physicochemical stability of the tofu matrix. Changes in water distribution are often accompanied by variations in water-holding capacity and textural properties in gel-type foods (32, 33). In the present study, the time-dependent changes in A_21_ and A_22_ occurred concurrently with the decreases in moisture content, WHC, and textural properties described above, suggesting a close association between water redistribution and quality deterioration. Similar studies have shown that changes in water distribution are closely related to texture deterioration of gel-type foods (34). Therefore, the LF-NMR results revealed distinct time-dependent shifts in water distribution among the different cold-chain treatments, suggesting that altered water status was associated with the deterioration of WHC and texture in tofu.

### 3.6 MRI

MRI can intuitively reflect the spatial distribution of proton signals associated with water inside food, and is an important method to evaluate changes in water status of gel-type foods (35). As shown in Figure 5, the proton density images of pre-packaged tofu under different cold chain logistics conditions showed obvious storage time-dependent changes. Among these, low-signal areas (blue) indicated relatively low proton signal intensity, while high-signal areas (red) corresponded to relatively high proton signal intensity, generally associated with a higher local water proton density (7). At the initial storage stage (0 d), samples in the CK, T1, and T2 groups all showed high signal intensity, with red areas dominating the images, indicating that the spatial distribution of water-related proton signals was relatively uniform. With the extension of storage time, the red areas in all groups gradually decreased and transformed into yellow and green areas, reflecting changes in the spatial distribution and relative intensity of water-related proton signals during storage. The degree of changes differed among samples under different logistics conditions, with the temperature fluctuation groups showing more obvious color changes, suggesting that temperature fluctuations were associated with greater changes in the spatial distribution of water-related signals. At 35 d, the high-signal areas of all three groups markedly decreased, shifting to an overall blue-green tone, indicating a general decline in proton signal intensity and a marked change in the spatial distribution of water-related signals. Among these, the T1 group showed the most obvious color changes, indicating that larger temperature fluctuations were associated with more pronounced changes in the spatial distribution of water-related signals; the T2 group showed changes between those of CK and T1. These MRI observations complemented the LF-NMR results: LF-NMR showed time-dependent changes in the relative proportions of water populations, whereas MRI visualized concurrent shifts in the spatial distribution of water-related proton signals. Similarly, Wang et al. (24) found that cold chain temperature fluctuations could significantly change the water status inside food, leading to decreased WHC and quality deterioration. In summary, MRI visualized differences in the spatial distribution of water-related proton signals inside tofu across logistics conditions. Stable cold-chain storage resulted in relatively small changes, whereas temperature fluctuation treatments, particularly T1, showed more pronounced alterations, consistent with the observed decreases in moisture content, WHC, and textural properties.

**Figure 5:**
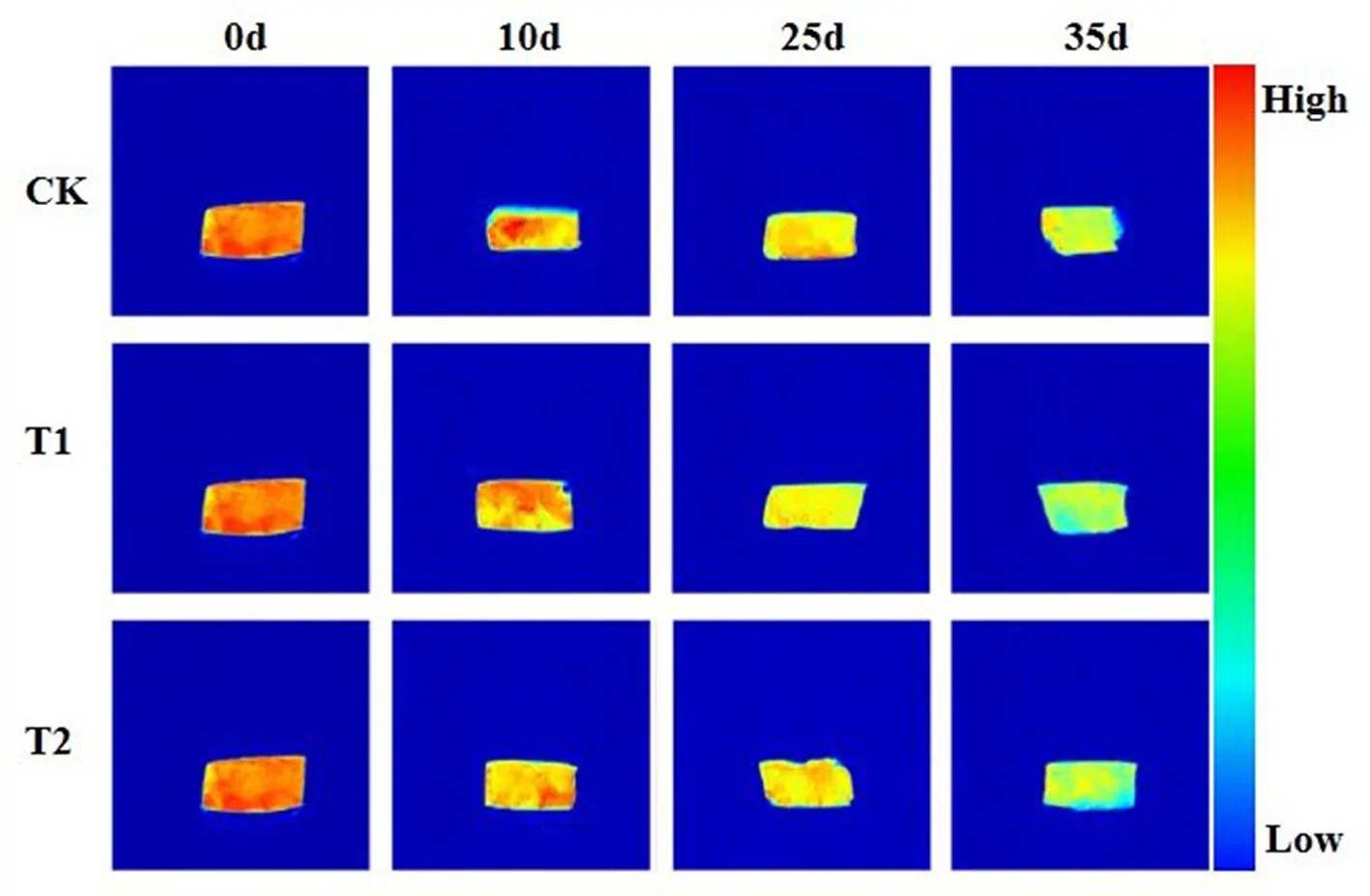
Proton density-weighted magnetic resonance imaging (MRI) pseudo-color maps of prepackaged tofu under different cold chain logistics conditions.

### 3.7 Electronic nose analysis

Electronic nose analysis can rapidly characterize changes in the overall odor profile during food storage through multiple gas-sensitive sensor response patterns, and can be used to evaluate the flavor deterioration trend of tofu under cold chain conditions (36). As shown in Figure 6, the electronic nose response values for pre-packaged tofu under different logistics modes changed significantly with storage time, and different temperature treatment groups exhibited different volatile odor response characteristics. At the initial state (0 d), the sensor response values of the CK, T1, and T2 groups were consistent, indicating that the initial overall odor response profiles of samples under different logistics conditions were similar. With the extension of storage time, the electronic nose response patterns of each treatment group gradually changed. Among these, differences between temperature fluctuation groups and the CK group began to appear after 10 d, indicating that shifts in the volatile odor profiles among cold-chain treatments became apparent. At 10 d of storage, the response value of sensor W5S in the T1 group reached 3.838, higher than that of the CK group (3.058) and T2 group (3.281), indicating a stronger response of W5S to the volatile compounds present in the T1 group at this stage. With further storage, the differences in odor responses among treatment groups further expanded. At 25 d, the sensor response patterns under different logistics conditions showed obvious differences, with the W5S response value of the CK group reaching 10.863, while those of the T1 and T2 groups reached 2.666 and 2.373, respectively, indicating that different temperature conditions were associated with distinct volatile odor profiles. At 35 d, all samples in each group showed relatively high responses to sensor W5S, with the CK, T1, and T2 groups reaching 10.307, 10.409, and 6.649, respectively, indicating pronounced changes in the volatile odor profile during the later stage of storage. Overall, electronic nose analysis indicated that temperature fluctuations were associated with more pronounced changes in the volatile odor characteristics of pre-packaged tofu during storage. Combined with the results of microbial growth, TVB-N accumulation, and biogenic amine formation described above, changes in the electronic nose response patterns occurred concurrently with shifts in these spoilage-related indicators. The electronic nose results provided a basis for subsequent analysis of key volatile compound changes using GC-IMS and GC-MS.

**Figure 6:**
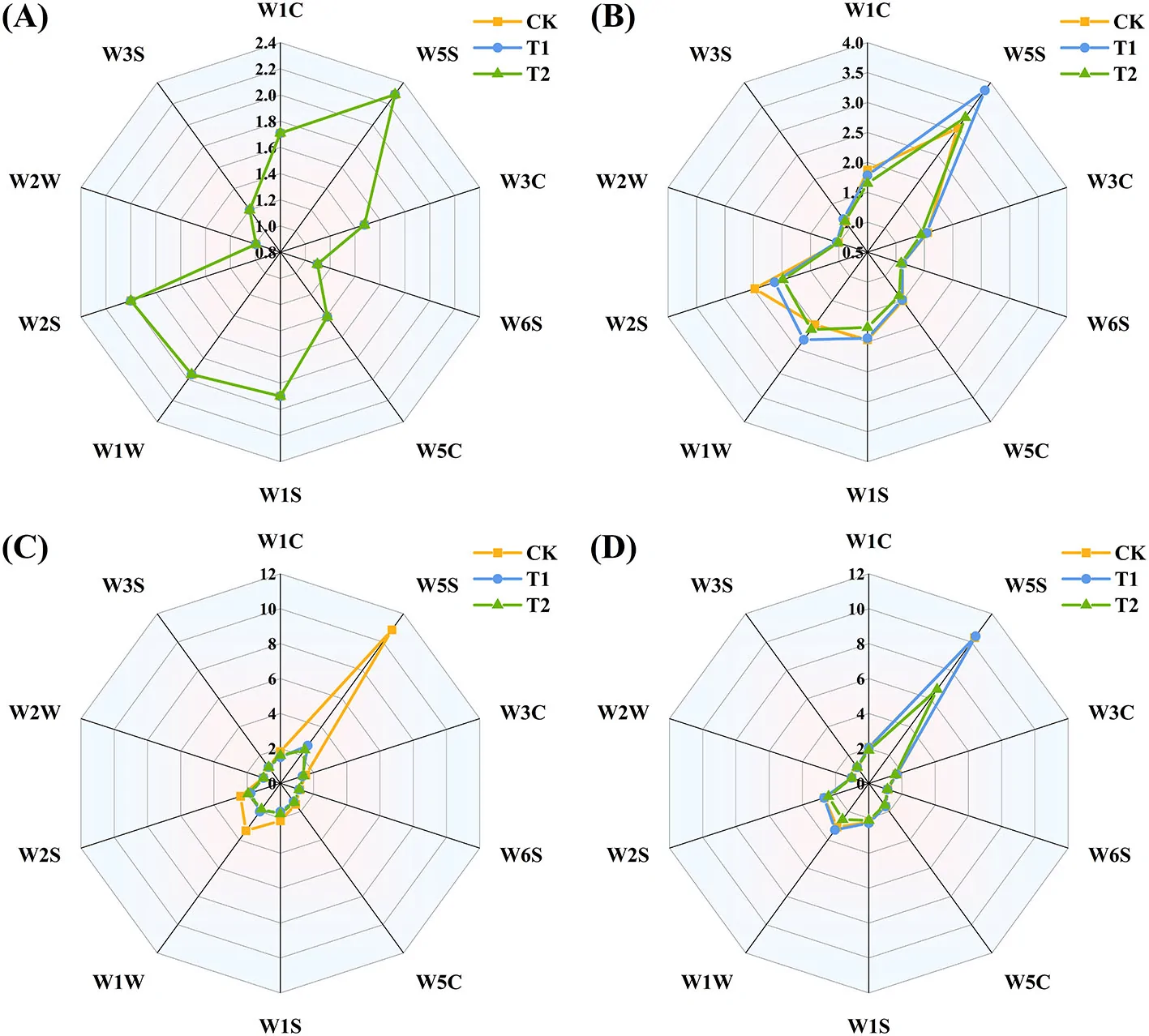
Radar plots of electronic nose sensor responses for prepackaged tofu under different cold chain logistics conditions. **(A)** 0 d; **(B)** 10 d; **(C)** 25 d; **(D)** 35 d.

### 3.8 GC-IMS analysis

#### 3.8.1 Three-dimensional spectra and difference maps

GC-IMS was used to analyze changes in volatile components of pre-packaged tofu under different cold chain logistics conditions, as shown in Figure 7. From the three-dimensional spectra (Figures 7A–C), it could be observed that samples in all groups showed abundant volatile signal peaks, indicating the presence of complex volatile components during tofu storage. Compared with samples at 0 d, the number and intensity of volatile signal peaks changed significantly after different cold chain logistics treatments, reflecting marked shifts in the overall volatile profiles during storage. With the extension of storage time, the degree of changes in volatile regions of the T1 and T2 groups was higher than that of the CK group, suggesting that temperature fluctuations were associated with more pronounced shifts in the volatile profile of tofu during storage.

**Figure 7:**
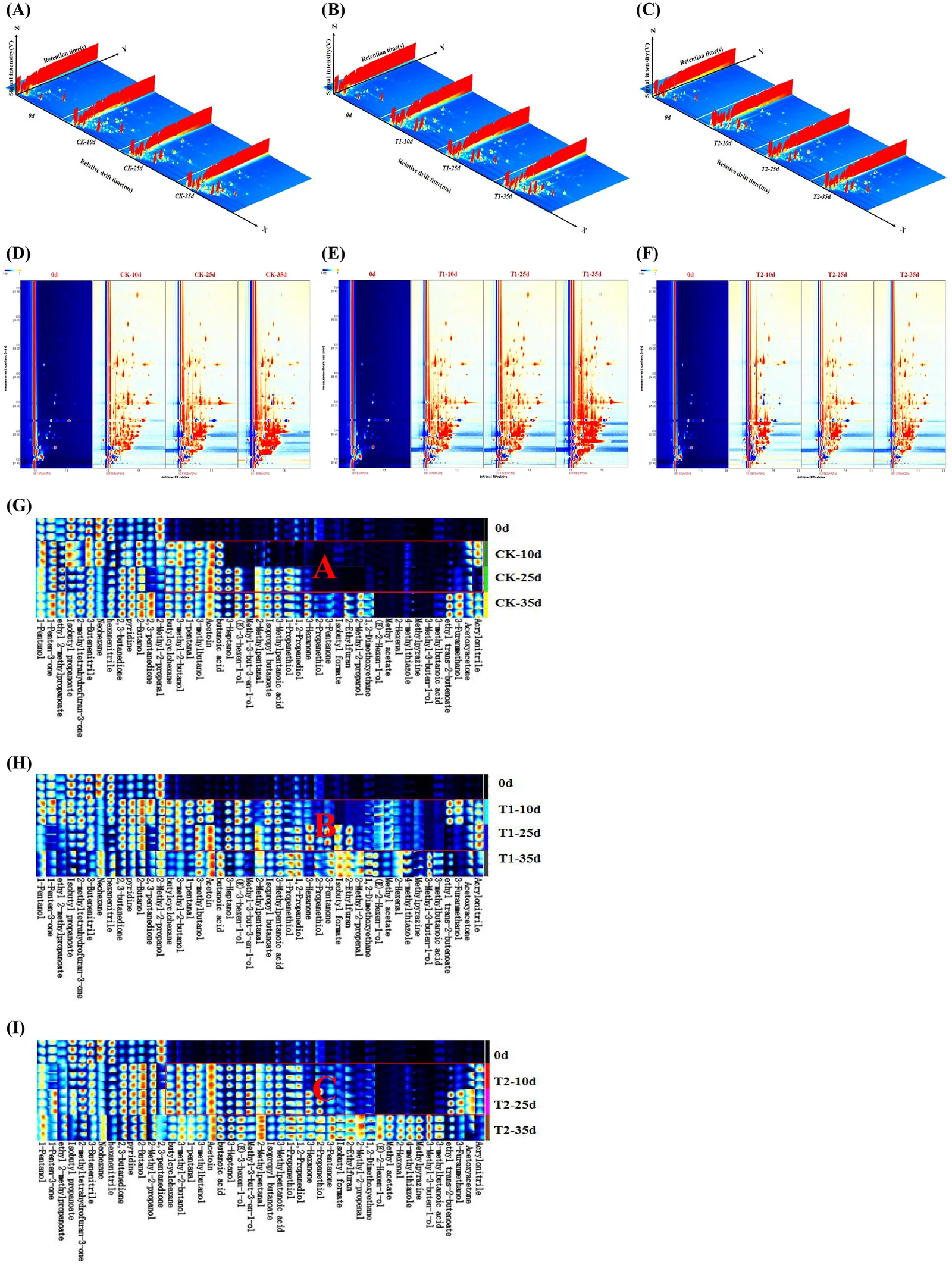
GC-IMS analysis of volatile compounds in prepackaged tofu under different cold chain logistics conditions. **(A–C)** Three-dimensional spectra of the CK, T1, and T2 groups, respectively; **(D–F)** two-dimensional difference contrast maps of the CK, T1, and T2 groups, respectively; **(G–I)** corresponding fingerprints of the CK, T1, and T2 groups, respectively.

The two-dimensional difference maps (Figures 7D–F) further reflected the dynamic changes of volatile components under different logistics conditions. With samples at 0 d as reference, red regions indicated increased signal intensity relative to the reference, while blue regions indicated a decreased signal intensity (37). With the extension of storage time, all groups showed different degrees of color changes, among which the T1 group showed a significant increase in red regions at the later stage, while the CK group changed relatively slowly. These results showed more pronounced changes in the overall volatile profile under temperature fluctuation conditions, particularly in the T1 group.

#### 3.8.2 Compositional changes of volatile compounds

A total of 45 volatile compounds were identified through GC-IMS database matching, mainly including alcohols, aldehydes, ketones, acids, esters, heterocyclic compounds, nitrogen-containing compounds, and hydrocarbons. The fingerprint spectra of volatile compounds under different cold chain logistics conditions showed obvious differences (Figures 7G–I), indicating that temperature fluctuations were associated with changes in the volatile composition of pre-packaged tofu during storage. Compared with the CK group, the response intensities of volatile compounds in the T1 and T2 groups were generally enhanced, with the T1 group showing the most pronounced increase, indicating that the larger temperature fluctuation treatment was associated with greater changes in volatile signal intensity. In the CK group (region A), the changes in volatile compounds during storage were relatively slow, with only partial compounds showing slight fluctuations, suggesting that stable low-temperature storage was associated with relatively smaller shifts in the volatile profile of tofu. In contrast, after temperature fluctuations, especially in the T1 group (region B), multiple volatile compounds showed enhanced signal intensities, mainly involving alcohols, aldehydes, ketones, and acids. Among these, the enhanced signals of alcohols (such as 3-methylbutanol, 3-heptanol, and 1-pentanol) may be related to lipid oxidation, fatty acid degradation, and microbial metabolism processes (38, 39). In addition, the enhanced signals of aldehydes (such as 1-pentanal, 2-methylpentanal, and 2-hexenal) were consistent with enhanced lipid oxidation. Aldehydes usually originate from oxidative degradation of unsaturated fatty acids, and their increase is closely related to the formation of off-odors such as grassy, oxidized, and beany flavors (40). Therefore, the greater responses of these oxidation-related aldehydes under temperature fluctuation conditions suggest enhanced lipid oxidation, which may contribute to the shift in tofu flavor from its original characteristics toward oxidative off-odors. In addition to oxidation-related volatiles, the enhancement of ketones in the T1 group may also be associated with changes in microbial metabolic activity. Among these, substances such as acetoin, 2,3-butanedione, and 2,3-pentanedione are usually associated with fermentation metabolic processes and can impart creamy or fermented odors to food (41). However, the continuous enhancement of their signals at the later storage stage might indicate enhanced microbial activity, consistent with the aforementioned trends of total bacterial counts, psychrophilic bacterial counts, and TVB-N. Meanwhile, the increase of short-chain acids such as butanoic acid, 3-methylbutanoic acid, and 3-methylpentanoic acid may be associated with enhanced degradation and microbial metabolic processes during storage. Compared with the T1 group, the degree of volatile changes in the T2 group (region C) was relatively lower, despite still showing increasing trends of some oxidation- and metabolism-related compounds. This indicated that a lower degree of temperature fluctuations may also affect tofu flavor components, but the changes were less pronounced than those observed under the larger temperature fluctuation treatment. Overall, the T1 group exhibited the most pronounced alteration in the volatile profile. The GC-IMS results showed higher signal intensities for several deterioration-related volatiles, including aldehydes, ketones, and acids, under temperature fluctuation conditions. These changes were consistent with enhanced lipid oxidation and microbial activity and may contribute to the deterioration of flavor quality in pre-packaged tofu.

### 3.9 HS-SPME-GC/MS analysis

#### 3.9.1 Compositional changes of volatile compounds

To further clarify the changes in volatile compound composition of tofu under different cold chain logistics conditions, GC-MS technology was used to detect and analyze samples in each group, and a total of 50 volatile compounds were identified, mainly including alcohols, aldehydes, ketones, acids, esters, hydrocarbons, heterocyclic compounds, and aromatic compounds (Table 3). The composition and relative contents of volatile compounds differed significantly under different logistics conditions, indicating that temperature fluctuations altered the volatile profiles of tofu during storage. Among these, alcohols were the most abundant, with 11 compounds detected, and were an important component of the volatile profile of tofu. Alcohols mainly originate from lipid oxidation, fatty acid degradation, and microbial metabolism processes (38). Among these, ethanol, 1-hexanol, and 1-octen-3-ol had relatively high contents, presenting alcoholic, grassy, fatty, and mushroom-like odors respectively, which are important contributors to the basic flavor of tofu. 1-Octen-3-ol, a typical lipid oxidation product, has a distinct mushroom-like and earthy odor, and its content changes can reflect the degree of lipid oxidation during storage (42). With the extension of storage time, some alcohols in temperature fluctuation groups changed significantly, suggesting that these shifts may be associated with lipid oxidation and microbial metabolism. Aldehydes were also an important part of the volatile composition of tofu, with 13 compounds detected. Aldehydes are usually formed by oxidative cleavage of unsaturated fatty acids, and are important markers of lipid oxidation during food storage (40). Among these, hexanal, nonanal, (E)-2-nonenal, and (E)-2-hexenal have grassy, beany, oxidized fatty and pungent odors, and are closely related to the off-flavors generated during tofu storage. In particular, hexanal is considered a typical contributor to oxidative off-odor in plant-based protein products, and its accumulation usually indicates enhanced lipid oxidation reactions (43). The greater abundance of oxidation-related aldehydes under temperature fluctuation conditions was consistent with the higher TBARS values, suggesting enhanced lipid oxidation.

**Table 3:** Volatile compounds identified in prepackaged tofu under different cold chain logistics conditions by HS-SPME- GC/MS.

Compounds	CAS	Odorant description	Semi-quantitative content (μg/kg)									
			0 d	CK-10 d	CK-25 d	CK-35 d	T1-10 d	T1-25 d	T1-35 d	T2-10 d	T2-25 d	T2-35 d
**Alcohols** **(****11****)**			1,118.39 ± 9.01	982.39 ± 12.88	1,080.75 ± 10.87	927.68 ± 3.75	1170.79 ± 6.43	1,014.31 ± 10.6	1,808.95 ± 6.29	660.3 ± 12.91	338.88 ± 5.34	496.19 ± 2.06
Ethanol	64-17-5	Alcoholic odor, fermented aroma	369.11 ± 4.74	279.05 ± 4.52	269.93 269.93 2.86	245.25 1.99	236.73 1.68	193.17 ± 2.91	329 ± 2.61	395.63 ± 4.47	309.26 ± 3.72	107.58 ± 3.08
1-Hexanol	111-27-3	Green, fatty, and slightly fruity odor	176.38 ± 1.92	156.43 ± 6.09 6.09	342.3 3.31	281.96 4.03	371.95 ± 3.58	220.08 ± 5.39	532.95 ± 2.66	182.11 ± 4.08	29.62 ± 5.27	266.85 ± 3.88
1-Octen-3-ol	3391-86-4	Mushroom-like, earthy, and fatty odor	572.9 ± 3.91	420.51 ± 3.33	408.92 ± 4.13	342.81 ± 0.99	514.95 ± 3.38	535.62 ± 2.52	783.91 ± 3.29	ND	ND	ND
2-Nonanol	628-99-9	Sweet, waxy, and fatty odor	ND	ND	3.16 ± 0.42	ND	ND	ND	3.68 ± 0.35	ND	ND	ND
1-Octanol	111-87-5	Citrus-like, fatty, and green odor	ND	50.38 ± 3.36	33.85 ± 1.81	ND	ND	ND	35.92 ± 0.21	ND	ND	35.69 ± 2.18
1-Nonanol	143-08-8	Waxy, floral, and fatty odor	ND	ND	ND	51.47 ± 4.46	ND	ND	79.77 ± 0.57	40.39 ± 5.34	ND	66.11 ± 4.81
(E)-2-Nonen-1-ol	31502-14-4	Green, fatty, and aldehydic odor	ND	ND	ND	ND	ND	ND	25.85 ± 1.04	ND	ND	ND
(E)-2-Octen-1-ol	18409-17-1	Green, mushroom-like, and fatty odor	ND	ND	ND	ND	47.16 ± 2.26	45.89 ± 0.15	ND	42.17 ± 1.57	ND	ND
(E)-2-Decen-1-ol	22104-80-9	Waxy, fatty, and vegetable oil-like odor	ND	ND	16.53 ± 2.35	ND	ND	19.56 ± 0.42	ND	ND	ND	9.41 ± 0.96
3-Decyn-2-ol	69668-93-5	Alcoholic and fatty odor	ND	76.02 ± 1.99	ND	ND	ND	ND	ND	ND	ND	ND
Benzyl alcohol	100-51-6	Floral, sweet, and almond-like odor	ND	ND	6.06 ± 0.38	6.2 ± 2.66	ND	ND	17.87 ± 0.77	ND	ND	10.56 ± 2.72
**Aldehydes** **(****13****)**			1,071.48 ± 1.05	821.29 ± 8.75	371.64 ± 7.39	481.3 ± 9.25	834.97 ± 3.52	944.9 ± 13.64	237.22 ± 1.03	677.74 ± 1.93	1,125.15 ± 11.86	310.78 ± 7.85
(E)-2-Heptenal	18829-55-5	Green, oxidative fatty, and pungent odor	ND	84.44 ± 5.57	63.39 ± 0.82	120.25 ± 2.68	85.95 ± 1.21	81.02 ± 4.67	63.64 ± 5.23	107.57 ± 5.04	144.29 ± 2.77	57.42 ± 1.46
Nonanal	124-19-6	Citrus-like, fatty, and waxy odor	263.75 ± 4.98	221.19 ± 3.85	145.74 6	109.3 2.99	281.94 4.6	276.71 5.28	52.62 ± 1.92	196.76 ± 2.22	381.03 ± 5.83	103.15 ± 3.3
(2E,4E)-Heptadienal	881395-00-0	Fatty, green, and lipid oxidation odor	12.73 ± 2.79	ND	9.06 ± 2.27	28.91 ± 5.45	8.48 ± 2.74	10.35 ± 1.71	16.6 ± 1.47	ND	12.82 ± 2.55	12.05 ± 2.82
Benzaldehyde	100-52-7	Bitter almond-like and sweet odor	62.87 ± 5.2	47 ± 6.3	13.55 ± 0.41	29.77 ± 3.58	67.17 ± 5.06	106.32 ± 4.71	23.82 ± 2.72	78.47 ± 2.87	159.54 ± 1.46	9.56 ± 0.41
(E)-2-Nonenal	18829-56-6	Cucumber-like, green, and oxidative fatty odor	87.47 ± 4.6	61.66 ± 4.62	42.85 ± 1.35	50.55 ± 1.16	84.02 ± 4.79	80.03 ± 1.21	66.34 ± 0.9	80.19 ± 3.28	49.67 ± 1.81	41.99 ± 2.37
(2E,4E)-Decadienal	25152-84-5	Fried, fatty, and nutty odor	140.07 ± 5.32	85.7 ± 3.47	44.88 ± 3.33	69.33 ± 1.63	ND	109.32 ± 2.34	14.2 ± 2.19	ND	ND	59.24 ± 1.34
Hexanal	66-25-1	Green, beany, and oxidative fatty odor	420.73 ± 6.09	182.15 ± 4.63	ND	ND	192.9 ± 3.66	216.73 ± 3.23	ND	59.64 ± 1.86	250.16 ± 5.04	ND
(E)-2-Hexenal	6728-26-3	Green, apple-like fruity, and pungent odor	48.41 ± 4.52	34.03 ± 0.31	ND	ND	46.58 ± 2.3	ND	ND	49.85 ± 0.89	ND	27.36 ± 1.75
(E)-2-Decenal	3913-81-3	Fatty and orange peel-like odor	35.45 ± 2	25.69 ± 1.51	ND	ND	47.54 ± 4.06	28.21 ± 4.5	ND	32.73 ± 1.68	51.03 ± 3.02	
Octanal	124-13-0	Citrus-like and sweet orange odor	ND	ND	ND	ND	20.38 ± 2.9	36.21 ± 1.14	ND	ND	ND	ND
(E)-2-Octenal	2548-87-0	Green, fatty, and nutty odor	ND	62.92 ± 2.19	52.17 ± 1.52	51.78 ± 1.47	ND	ND	ND	72.52 ± 1.47	56.4 ± 2.45	ND
(E)-2-Undecenal	53448-07-0	Waxy, fatty, and orange peel-like odor	ND	16.52 ± 5.33	ND	21.41 ± 1.86	ND	ND	ND	ND	ND	ND
(8Z)-2-Undecenal	147159-49-7	Fatty and waxy odor	ND	ND	ND	ND	ND	ND	ND	ND	20.2 ± 0.36	ND
**Ketones** **(****2****)**			ND	ND	190.16 ± 3.06	229.49 ± 5.27	107.55 ± 1.88	67.97 6.17	137.61 2.94	74.18 ± 4.42	16.49 ± 2.31	171.6 ± 6.54
Acetoin	513-86-0	Creamy, buttery, and sweet odor	ND	ND	190.16 ± 3.06	229.49 ± 5.27	107.55 ± 1.88	67.97 ± 6.17	113.1 ± 4.31	74.18 ± 4.42	16.49 ± 2.31	163.7 ± 4.91
2-Heptanone	821-55-6	Fruity, cheesy, and slightly spicy odor	ND	ND	ND	ND	ND	ND	24.51 ± 1.37	ND	ND	7.9 ± 1.63
**Acids** **(****7****)**			85.52 ± 4.14	170.65 ± 2.87	71.34 ± 3.17	154.99 154.99 4.97	129.52 5.36	111.22 ± 2.58	626.59 ± 9.73	122.07 ± 4.11	235.41 ± 2.59	594.98 ± 3.24
Butyric acid	107-92-6	Rancid, cheesy, and sweaty odor	ND	ND	ND	ND	ND	ND	387.84 ± 3.84	ND	32.18 ± 0.07	21.97 ± 2.45
Hexanoic acid	142-62-1	Cheesy, fatty acid-like, and pungent acidic odor	ND	ND	42.09 ± 2.42	49.64 1.28	50.51 1.83	66.7 ± 5.09	151.4 ± 3.05	55.04 ± 1.62	91.11 ± 2.56	65.04 ± 0.16
Octanoic acid	124-07-2	Fatty, cheesy, and waxy odor	ND	ND	ND	61.57 ± 3.26	ND	26.92 ± 1.85	56.45 ± 2.68	39.59 ± 5.73	32.98 ± 2.71	ND
Nonanoic acid	112-05-0	Fatty acid-like and oily odor	85.52 ± 4.14	158.9 ± 1.96	29.25 ± 0.75	43.77 ± 2.18	79.01 ± 3.81	17.59 ± 3.41	30.91 ± 0.16	27.44 ± 1.77	79.14 ± 2.68	16.88 ± 1.45
Undecanoic acid	112-37-8	Waxy and fatty odor	ND	11.75 ± 0.91	ND	ND	ND	ND	ND	ND	ND	ND
Acetic acid	64-19-7	Vinegar-like and acidic odor	ND	ND	ND	ND	ND	ND	ND	ND	ND	449.9 ± 1.96
Decanoic acid	334-48-5	Oily and fatty acid-like odor	ND	ND	ND	ND	ND	ND	ND	ND	ND	41.18 ± 1.76
**Esters** **(****6****)**			ND	176.72 ± 5.33	ND	ND	103.57 ± 1.09	61.03 ± 6.58	208.4 ± 7.1	ND	14.64 ± 3.33	19.78 ± 1.2
Vinyl acetate	108-05-4	Fruity, sweet, and slightly ether-like odor	ND	ND	ND	ND	ND	ND	160.95 ± 0.03	ND	ND	ND
Butyl butyrate	109-21-7	Pineapple-like fruity and sweet odor	ND	ND	ND	ND	ND	ND	16.58 ± 3.96	ND	ND	ND
Hexyl acetate	142-92-7	Apple-like and banana-like fruity odor	ND	ND	ND	ND	ND	18.63 ± 5.07	30.87 ± 3.16	ND	ND	ND
Ethyl acetate	141-78-6	Fruity, alcoholic, and solvent-like odor	ND	176.72 ± 5.33	ND	ND	103.57 ± 1.09	42.4 ± 1.62	ND	ND	ND	ND
Allyl 2-ethylbutyrate	7493-69-8	Fruity and pineapple-like odor	ND	ND	ND	ND	ND	ND	ND	ND	ND	11.54 ± 0.01
Dimethyl glutarate	1119-40-0	Sweet and ester-like odor	ND	ND	ND	ND	ND	ND	ND	ND	14.64 ± 3.33	8.24 ± 1.19
**Hydrocarbons** **(****7****)**			ND	ND	19.94 ± 1.6	152.89 ± 4.54	61.57 ± 6.94	53.84 ± 3.06	ND	ND	48.61 ± 1.61	27.38 ± 5.21
1-Nonene	124-11-8	Waxy and slightly petroleum-like odor	ND	ND	ND	ND	36.8 ± 6.39	53.84 ± 3.06	ND	ND	ND	ND
Tridecane	629-50-5	Waxy and hydrocarbon-like odor	ND	ND	ND	ND	24.76 ± 0.78	ND	ND	ND	ND	ND
Tetradecane	629-59-4	Waxy and alkane-like odor	ND	ND	19.94 ± 1.6	50.87 ± 2.91	ND	ND	ND	ND	41.21 ± 0.56	27.38 ± 5.21
Undecane	1120-21-4	Waxy and oily odor	ND	ND	ND	37.15 ± 0.11	ND	ND	ND	ND	ND	ND
Pentadecane	629-62-9	Waxy and alkane-like odor	ND	ND	ND	12.3 ± 1.93	ND	ND	ND	ND	ND	ND
Hexadecane	544-76-3	Waxy and oily odor	ND	ND	ND	ND	ND	ND	ND	ND	7.39 ± 1.05	ND
2-Bromododecane	13187-99-0	Hydrocarbon-like odor	ND	ND	ND	52.57 ± 1.88	ND	ND	ND	ND	ND	ND
**Heterocyclic compounds** **(****2****)**			216.54 ± 2.12	157.29 ± 3.3	110.45 ±± 0.94	164.12 ± 0.76	204.81 ± 0.57	186.45 ± 1.09	268.65 ± 2.48	217.32 ± 6.76	214.15 ± 4.99	124.73 ± 6.06
2-Pentylfuran	3777-69-3	Beany, green, and oxidative fatty odor	160.38 ± 4.26	107.43 ± 1.4	94.11 1.33	106.32 ± 3.35	124.26 ± 5.72	104.86 ± 2.05	213.64 ± 2.28	150.92 ± 4.15	214.15 ± 4.99	97.61 ± 3.47
Maltol	118-71-8	Caramel-like, sweet, and roasted odor	56.17 ± 2.28	49.86 ± 3.88	16.34 2.28	57.8 ± 3.89	80.55 ± 6.28	81.59 ± 1.05	55.02 ± 4.76	66.4 ± 3.13	ND	27.12 ± 2.64
**Aromatic hydrocarbons** **(****3****)**			ND	33.7 ± 4.63	39.64 ± 2.25	67.22 ± 2.68	ND	76.34 ± 2.84	89.37 ± 5.23	34.55 ± 1.7	26.15 ± 2.02	
o-Xylene	95-47-6	Aromatic hydrocarbon-like and solvent-like odor	ND	ND	ND	ND	ND	ND	89.37 ± 5.23	ND	ND	ND
m-Xylene	108-38-3	Aromatic hydrocarbon-like, sweet, and solvent-like odor	ND	ND	ND	ND	ND	76.34 ± 2.84	ND	ND	ND	ND
Ethylbenzene	100-41-4	Aromatic hydrocarbon-like and gasoline-like odor	ND	33.7 ± 4.63	39.64 ± 2.25	67.22 ± 2.68	ND	ND	ND	34.55 ± 1.7	26.15 ± 2.02	ND

ND, means not detected due to the concentration of given compound was below the detection limit; CAS, Chemical Abstracts Service.

In addition to oxidation-related substances, changes in ketones, acids, and esters further reflect metabolic changes during tofu storage. Ketones mainly included acetoin and 2-heptanone, among which acetoin has creamy and sweet aromas and its formation is closely related to microbial metabolism processes (41); while 2-heptanone has fruity and cheesy odors, and is usually associated with fatty acid oxidation and microbial action (44). Acids mainly included butyric acid, hexanoic acid, and nonanoic acid, and their content increase is usually related to lipid hydrolysis and microbial metabolism (45), which can produce rancid, cheesy, and pungent acidic odors, serving as important flavor indicators of tofu quality deterioration. In addition, esters such as ethyl acetate, hexyl acetate, and vinyl acetate have fruity, sweet, and slightly ethereal characteristics, and their moderate presence may contribute fruity, sweet, and slightly ethereal notes to the volatile profile, but their changes may also be associated with alcohol- and acid-related processes during storage. Although heterocyclic compounds (2-pentylfuran, maltol) and aromatic compounds (ethylbenzene, xylene-type substances) were relatively low in content, their formation has been associated with lipid oxidation, sugar-amino reactions, and other complex processes (46). Overall, the GC-MS results indicated that different cold chain logistics conditions significantly affected the volatile compound composition of tofu. Volatile substances changed relatively slowly under stable cold chain conditions, whereas temperature fluctuations were associated with greater increases in oxidation-related volatiles, acids, and potential microbial metabolites, contributing to a gradual shift in the volatile profile toward oxidation- and spoilage-related characteristics.

#### 3.9.2 Key differential volatile compounds identified by OPLS-DA

To further screen the key compounds contributing to volatile differences in tofu under different cold chain logistics conditions, an OPLS-DA model was established based on the GC-MS results. As shown in Figure 8A, samples from different treatment groups showed obvious separation trends in the model space, indicating clear differences in volatile profiles among samples subjected to different cold-chain conditions. The OPLS-DA model yielded R^2^X(cum), R^2^Y(cum), and Q^2^(cum) values of 0.998, 0.999, and 0.998, respectively, indicating high goodness of fit and predictive ability. Given the high R^2^Y(cum) and Q^2^(cum) values, potential model overfitting was further assessed using permutation testing and CV-ANOVA. The model stability was further validated by 200 permutation tests (Figure 8B), with the results showing that the R^2^ intercept was 0.187 and the Q^2^ intercept was −0.727. The negative Q^2^ intercept suggested that no obvious overfitting was detected in the permutation validation. In addition, CV-ANOVA showed that the model was statistically significant (*F* = 467.606, *P* < 0.001), further supporting its validity.

**Figure 8:**
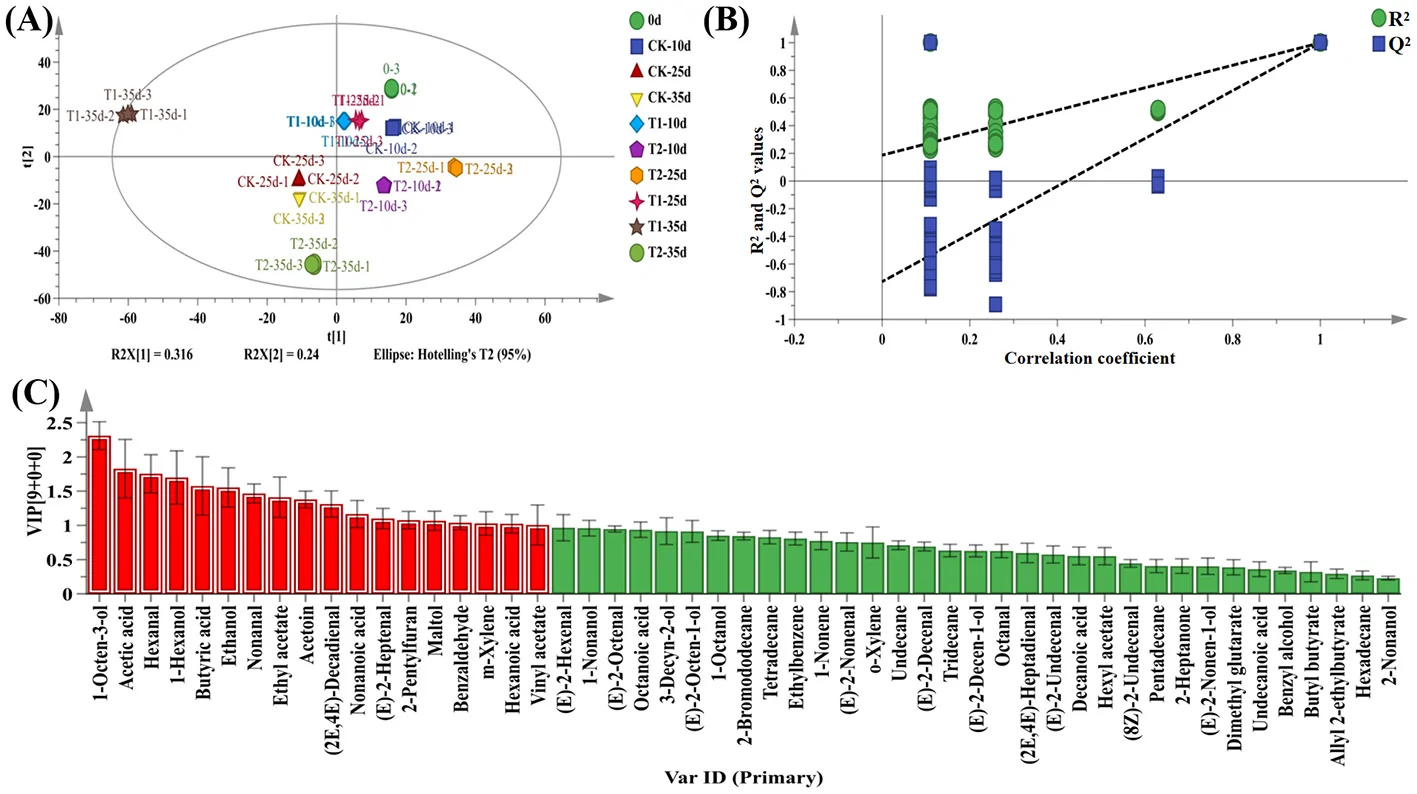
OPLS-DA model for differentiating volatile profiles of prepackaged tofu under different cold chain logistics conditions. **(A)** Score plot; **(B)** permutation test (*n* = 200); **(C)** VIP plot (VIP > 1).

Based on the variable importance in projection (VIP) values, key volatile compounds contributing to inter-group differences were further screened, and compounds with VIP values greater than 1 were considered important differential variables (Figure 8C). The results showed that a total of 18 potential differential volatile compounds were screened, mainly including 1-octen-3-ol, acetic acid, hexanal, 1-hexanol, butyric acid, ethanol, nonanal, ethyl acetate, acetoin, and (2E,4E)-decadienal. These compounds included volatiles commonly associated with lipid oxidation, microbial metabolism, and flavor transformation, suggesting that these processes may contribute to the differences observed among the cold-chain treatments. Among these, 1-octen-3-ol and hexanal had relatively high VIP values and were important compounds affecting flavor differences among different treatment groups. 1-Octen-3-ol usually originates from the oxidation process of unsaturated fatty acids, has typical mushroom-like, earthy, and fatty odors, and is considered an important oxidation-related volatile in plant-based protein products (42); hexanal is mainly produced by oxidative cleavage of polyunsaturated fatty acids such as linoleic acid, has grassy, beany, and oxidized fatty odors, and is an important indicator for evaluating the oxidation degree of plant-based foods (47). Therefore, the changes in these compounds, together with the increased TBARS values, were consistent with enhanced lipid oxidation under temperature fluctuation conditions and may contribute to the development of oxidative off-odors. In addition, acids such as acetic acid, butyric acid, and hexanoic acid also had relatively high contributions. Short-chain fatty acids mainly originate from lipid hydrolysis and microbial metabolism processes, and their accumulation can produce rancid, sweaty, and pungent odors (48), serving as important manifestations of quality deterioration in tofu at the later storage stage. Among ketones, acetoin has creamy and sweet aromas, and its formation is closely related to microbial metabolism; its moderate presence can enrich food aroma, but its abnormal changes might reflect enhanced microbial activity during storage. Meanwhile, esters such as ethyl acetate have fruity and sweet characteristics, and their changes may reflect alterations in alcohol- and acid-related processes during storage. In summary, OPLS-DA combined with VIP analysis screened key contributing substances for volatile differences in tofu under different cold chain logistics conditions. Among these, lipid oxidation-related compounds (1-octen-3-ol, hexanal, nonanal), microbial metabolism-related substances (acetic acid, butyric acid, acetoin), and some ester volatiles were identified as key contributors to the observed differences in volatile profiles among cold-chain treatments.

### 3.10 Proposed pathways associated with quality deterioration

Based on the above results and previous studies, a schematic representation of the potential pathways associated with quality deterioration of pre-packaged tofu under cold-chain temperature fluctuations was proposed (Figure 9). The repeated heating-cooling cycles during cold chain transportation may reduce the inhibitory effect of continuous low-temperature storage on microbial growth, thereby favoring greater microbial proliferation. Previous studies have shown that temperature fluctuations can alter microbial activity and accelerate the degradation of nutrients such as proteins and lipids in refrigerated foods (19). In this study, the temperature fluctuation groups showed higher total bacterial counts, psychrophilic bacterial counts, and TVB-N and biogenic amine contents, supporting an association between enhanced microbial proliferation and greater protein degradation, as reflected by the accumulation of spoilage-related metabolites, under fluctuating temperature conditions. Meanwhile, the temperature fluctuation treatments also showed higher TBARS values and greater increases in oxidation-related volatile compounds, indicating more pronounced lipid oxidation during storage. Lipid oxidation is known to generate secondary volatile products, including aldehydes and ketones, and compounds such as hexanal and nonanal are commonly regarded as oxidation-related markers associated with fatty, rancid, and off-odors (40). Accordingly, the increases in TBARS and oxidation-related volatile compounds observed in the present study were consistent with greater oxidative deterioration under temperature fluctuation conditions.

**Figure 9:**
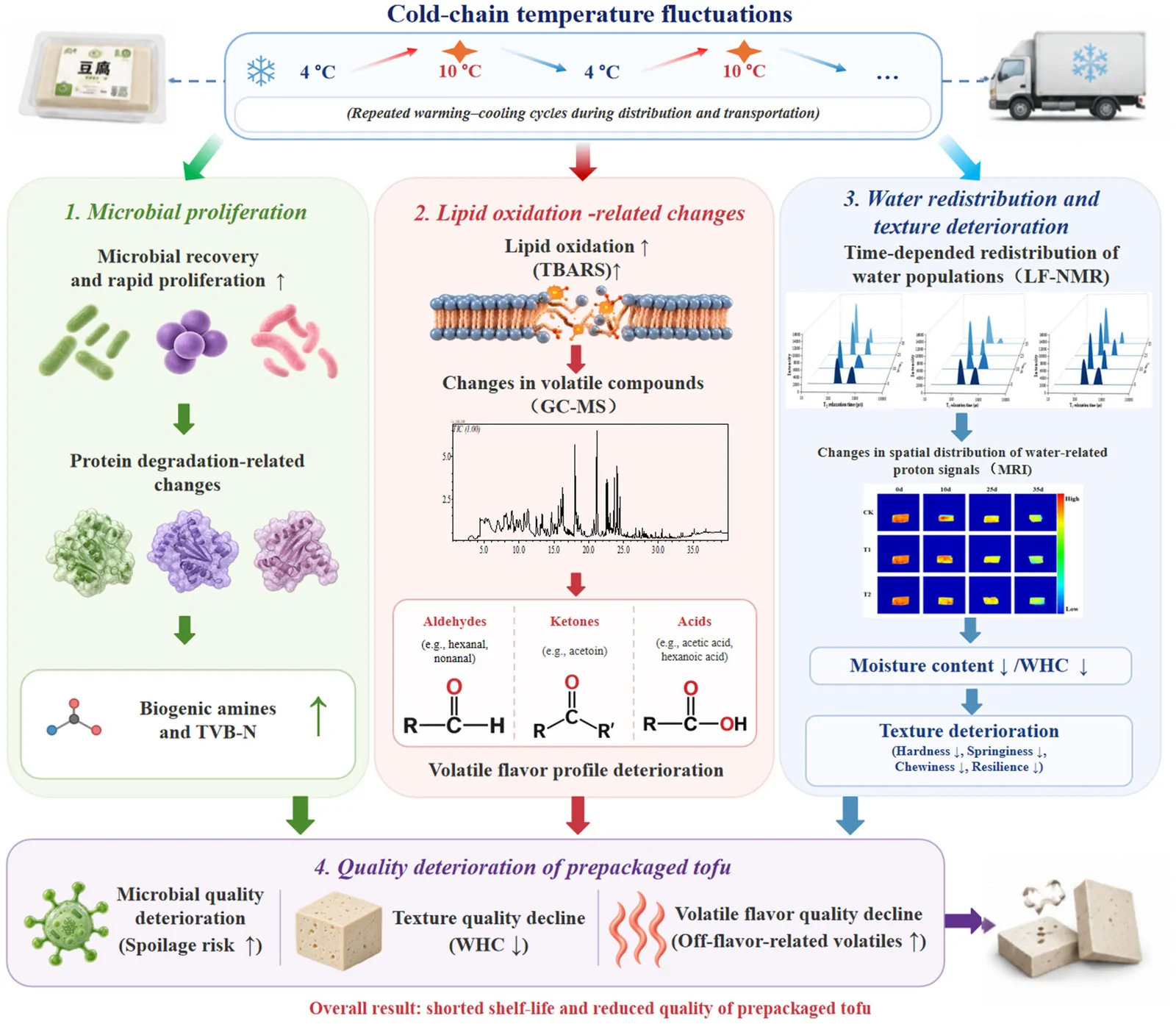
Proposed mechanism of temperature fluctuation-induced quality deterioration in prepackaged tofu during cold chain logistics.

In addition to these microbial and lipid oxidation-related effects, temperature fluctuations were associated with pronounced alterations in the water status and textural properties of tofu. In this study, temperature fluctuations were associated with time-dependent shifts in water distribution, accompanied by decreases in moisture content, WHC, and textural properties, including hardness, springiness, and chewiness, consistent with a close relationship between water redistribution and textural deterioration. These effects were more pronounced in the T1 group, which was subjected to more frequent temperature fluctuations, consistent with its greater microbial proliferation, higher spoilage-related physicochemical indices, and more pronounced changes in volatile profiles. In summary, cold-chain temperature fluctuations were associated with concurrent microbial proliferation; protein degradation (reflected by the accumulation of TVB-N and biogenic amines); lipid oxidation; time-dependent shifts in water distribution; reduced moisture content, WHC, and textural properties; and pronounced alterations in volatile profiles. Collectively, these experimental findings and their associations provide a basis for the proposed quality-deterioration pathways shown in Figure 9. However, Figure 9 should be interpreted as a conceptual framework based on the measured quality indicators and their associations rather than direct evidence of specific molecular or enzymatic mechanisms, which require further experimental verification.

## 4 Conclusion

This study systematically investigated the effects of temperature fluctuations during cold-chain logistics on quality changes and flavor deterioration of pre-packaged tofu. Temperature fluctuations were associated with greater microbial proliferation, increased TVB-N, biogenic amine, and TBARS levels, time-dependent shifts in water distribution, decreased water-holding capacity and textural properties, and pronounced alterations in volatile profiles, with the T1 treatment generally showing the greatest deterioration. GC-IMS and GC-MS analyses further revealed marked changes in oxidation- and spoilage-related volatile compounds, including aldehydes, ketones, and acids, under fluctuating temperature conditions. Collectively, these results indicate that temperature fluctuations accelerate the overall quality deterioration of pre-packaged tofu and suggest associations among microbial proliferation, lipid oxidation, water redistribution, texture deterioration, and volatile flavor changes, although the specific molecular and enzymatic mechanisms require further verification. It should be noted that the present findings were specifically obtained from heat-treated tofu containing the biopreservative chitosan oligosaccharides. The formulation and processing conditions of this product may influence its response to temperature fluctuations; therefore, caution should be exercised when generalizing the quantitative findings to other tofu products. Future studies should validate these responses in tofu products with different formulations, processing conditions, and packaging systems. Overall, this study provides experimental evidence for understanding quality deterioration under dynamic cold-chain conditions and offers a basis for optimizing temperature management during the storage and distribution of pre-packaged tofu. In addition, sensory evaluation was not conducted in the present study; future studies should combine sensory evaluation at appropriate storage stages with instrumental analyses to further clarify the relationship between volatile flavor changes and sensory acceptability.

## Data Availability

The original contributions presented in the study are included in the article/supplementary material, further inquiries can be directed to the corresponding author.
